# RNA m6A methylation orchestrates cancer growth and metastasis via macrophage reprogramming

**DOI:** 10.1038/s41467-021-21514-8

**Published:** 2021-03-02

**Authors:** Huilong Yin, Xiang Zhang, Pengyuan Yang, Xiaofang Zhang, Yingran Peng, Da Li, Yanping Yu, Ye Wu, Yidi Wang, Jinbao Zhang, Xiaochen Ding, Xiangpeng Wang, Angang Yang, Rui Zhang

**Affiliations:** 1 The State Key Laboratory of Cancer Biology, Department of Immunology, Fourth Military Medical University, Xi’an, Shaanxi China; 2 Henan Key Laboratory of Immunology and Targeted Therapy, School of Laboratory Medicine, Xinxiang Medical University, Xinxiang, Henan China; 3 The State Key Laboratory of Cancer Biology, Department of Biochemistry and Molecular Biology, Fourth Military Medical University, Xi’an, Shaanxi China; 4 Henan Collaborative Innovation Center of Molecular Diagnosis and Laboratory Medicine, School of Laboratory Medicine, Xinxiang Medical University, Xinxiang, Henan China; 5 Key Laboratory of Infection and Immunity of CAS, CAS Center for Excellence in Biomacromolecules, Institute of Biophysics, University of Chinese Academy of Sciences, Chinese Academy of Sciences, Beijing, China; 6 The Second Ward of Gynecological Tumor, Shaanxi Provincial Tumor Hospital, Xi’an, Shaanxi China; 7 Department of Thyroid, Breast and Vascular Surgery, Xijing Hospital, Fourth Military Medical University, Xi’an, China; 8 Department of Experimental Surgery, Xijing Hospital, Fourth Military Medical University, Xi’an, Shaanxi China

**Keywords:** Cancer microenvironment, Epigenetics in immune cells

## Abstract

N6-methyladenosine (m6A) is a reversible mRNA modification that has been shown to play important roles in various biological processes. However, the roles of m6A modification in macrophages are still unknown. Here, we discover that ablation of Mettl3 in myeloid cells promotes tumour growth and metastasis in vivo. In contrast to wild-type mice, Mettl3-deficient mice show increased M1/M2-like tumour-associated macrophage and regulatory T cell infiltration into tumours. m6A sequencing reveals that loss of METTL3 impairs the YTHDF1-mediated translation of SPRED2, which enhances the activation of NF-kB and STAT3 through the ERK pathway, leading to increased tumour growth and metastasis. Furthermore, the therapeutic efficacy of PD-1 checkpoint blockade is attenuated in Mettl3-deficient mice, identifying METTL3 as a potential therapeutic target for tumour immunotherapy.

## Abstract (web summary)

N6-methyladenosine (m6A) is a reversible mRNA modification with important roles in cancer biology and immunoregulation. Here, the authors show that myeloid-specific deletion of Mettl3, the catalytic subunit of the methyltransferase complex, promotes tumor growth and metastasis in preclinical tumor models, influencing macrophage reprogramming and attenuating PD-1 blockade.

## Introduction

Most tumours shape the tumour microenvironment to promote tumour growth by recruiting stromal cells, such as tumour-associated macrophages (TAMs)^1^, myeloid-derived suppressor cells (MDSCs)^2^ and regulatory T cells (Tregs)^3,4^, all of which are associated with a poor patient prognosis. An increasing number of studies have shown that the tumour microenvironment reprograms the gene expression of TAMs to reshape and maintain immunosuppressive functions^5–8^. TAMs often exhibit an array of activation states. In general, they are skewed away from the “classically” activated antitumour phenotype (referred to as M1) and towards an “alternatively” activated protumour phenotype (M2). However, like macrophages in many other tissues, TAMs show remarkable functional plasticity and often express markers characteristic of both activation states^9,10^. Factors responsible for the establishment and maintenance of TAM functions are beginning to emerge^7^. In particular, proteins associated with the regulation of RNA m6A modification are interesting candidates.

m6A, as an mRNA modification, is abundant in nearly all eukaryotes^11^. METTL3, serving as a catalytic subunit of the methyltransferase complex, has been reported to participate in diverse biological processes, including spermatogenesis^12^, neurogenesis^13^, sex determination^14,15^, stem cell self-renewal and fate determination^16,17^. Moreover, aberrant m6A modifications are frequently found in cancers, such as lung and colon adenocarcinomas^18^, endometrial cancer^19^, breast cancer^20^, glioblastomas^21,22^, acute myeloid leukaemia^23,24^ and hepatocellular carcinoma^25^, and execute their progressive or repressive roles in different tumours depending on the molecular targets.

m6A modification has been associated with immunoregulation. A recent article on a mouse model reports that m6A-modified mRNAs encoding lysosomal cathepsins are recognized by YTHDF1 in dendritic cells (DCs), subsequently enhancing the translation of cathepsins and facilitating tumour growth and shortening host survival^26^. In CD4^+^ T cells, m6A levels are largely responsible for controlling naive T-cell homeostasis^27^. As most studies mainly focus on tumour-intrinsic oncogenic pathways, the potential roles of m6A modification in host antitumour immune responses are largely unknown.

In this work, we show that myeloid-specific deletion of METTL3, the catalytic subunit of the methyltransferase complex, enhances tumour growth and metastasis in mouse tumour models and attenuates PD-1 blockade therapy via influencing macrophage reprogramming. Our study illustrates that m6A modification in macrophages might represent a validated target for cancer therapy.

## Results

### Myeloid-specific deletion of Mettl3 promotes tumour growth and metastasis

To investigate the functions of m6A modification in macrophages, WT BMDMs (bone marrow-derived macrophages) were incubated with B16 cells or B16 cell culture medium. We found that the expression of *Mettl3* was significantly reduced, whereas no appreciable difference was noted in *Mettl14* levels (Supplementary Fig. 1a). Furthermore, western blot analysis also confirmed decreased METTL3 protein expression (Supplementary Fig. 1b). To further characterize *Mettl3* gene expression, we isolated TAMs (CD11b^+^F4/80^+^) from tumour tissue and BMDMs from the same host mice and determined that the levels of *Mettl3* transcripts in TAMs decreased during tumour progression (Supplementary Fig. 1c). Similar results were also obtained by immunofluorescence staining for METTL3 in TAMs and BMDMs from the same host mice (Supplementary Fig. 1c).

**Fig. 1:**
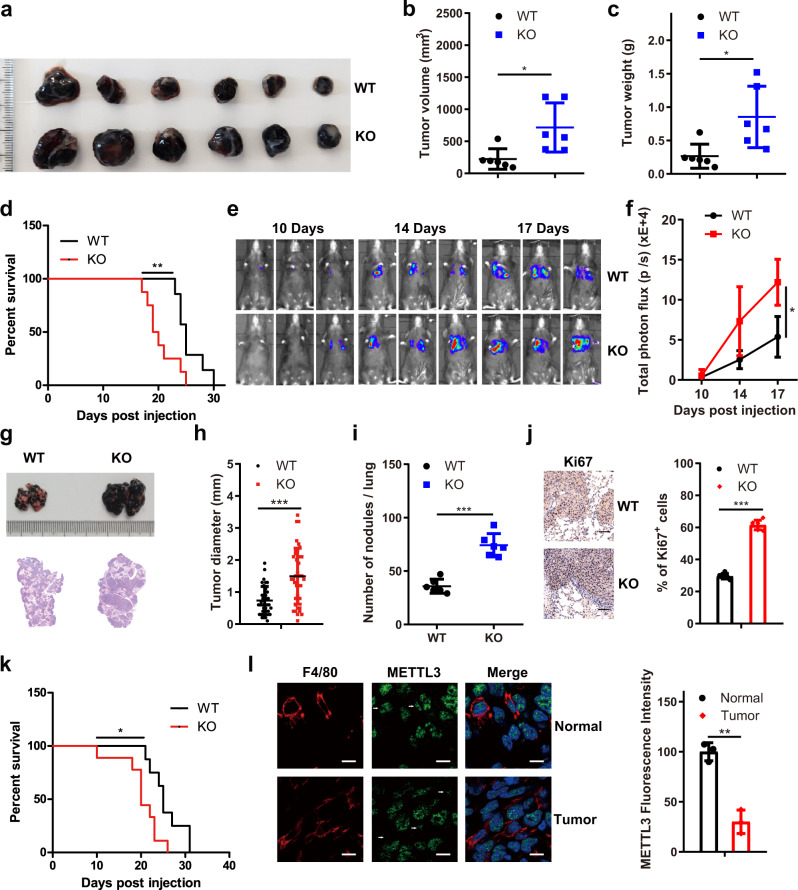
Myeloid-specific deletion of *Mettl3* in mice promotes B16 tumour growth and lung metastasis. **a**–**d** B16 cells were subcutaneously injected into WT and *Mettl3*-null (KO) mice. B16 tumours were dissected and photographed (**a**). Tumour volume (**b**) (*n* = 6 mice per group), tumour weight (**c**) (*n* = 6 mice per group) and mouse survival (**d**) were recorded (WT group, *n* = 7 mice; KO group, *n* = 8 mice). **e**, **f** In vivo imaging of mice was conducted following intraperitoneal administration of a substrate (D-luciferin). Representative fluorescence images are shown (**e**) and were quantified (**f**) (*n* = 3 mice per group). **g** Representative images of tumours in mice injected with B16 tumour cells via the tail vein (upper panel) and representative HE staining images of lung sections (lower panel) are shown. **h**, **i** Quantification of the lung tumour diameter (**h**) and metastatic nodules (**i**) of the mice evaluated in (**g**) (*n* = 6 mice per group) was performed. **j** Representative Ki67 staining images of tumour sections are shown (left panel). Quantification of Ki67 expression was performed (right panel) (*n* = 5 mice per group). Scale bars: 100 μm. **k** The survival of mice injected with B16 tumour cells via the tail vein was documented (*n* = 8 mice per group). **l** Immunofluorescence staining for F4/80 and METTL3 was performed to analyse expression in normal and tumour tissues (left panel). Quantification of the expression of METTL3 in macrophages was performed (right panel). The white arrows highlight F4/80^+^ cells *(n* = 3 mice per group). Scale bars, 10 μm. Data are expressed as the mean ± SD. *P* values were determined by a two-tailed *t*-test (**b**, **c**, **f**, **h**, **i**, **j** and **l**) and the Gehan–Breslow-Wilcoxon test (**d**, **k**). *P ≤ 0.05, **P < 0.01 and ***P < 0.001. The source data are provided as a Source data file.

We next asked whether *Mettl3* in myeloid cells regulates tumour progression. We crossed *Mettl3*^fl/fl^ mice with Lyz-cre mice to ablate *Mettl3* in the myeloid compartment, including in macrophages. Next, BMDMs and peritoneal macrophages (PMs) from *Mettl3*^fl/fl^Lyz2^+/+^ (WT) and *Mettl3*^fl/fl^Lyz2^cre/+^ (KO) mice were subjected to qRT-PCR and western blotting. The results demonstrated that the BMDMs from the KO mice had an ∼75% reduction in *Mettl3* mRNA expression and that PMs had an ∼80% reduction (Supplementary Fig. 1d, e), consistent with the reported ∼80% deletion efficiency in the Lyz-cre model^28^. Moreover, the m6A level of mRNAs in the KO BMDMs was lower than that in the WT BMDMs (Supplementary Fig. 1f). We next asked whether myeloid-specific deletion of *Mettl3* affects tumour growth. We injected B16 or LLC cells subcutaneously into syngeneic WT or KO mice. The KO mice exhibited significantly faster tumour growth than the WT mice, and the survival of these conditional *Mettl3* knockout mice was shortened (Fig. 1a–d and Supplementary Fig. 1g-j). Furthermore, to determine whether METTL3 can affect tumour metastasis, B16 or LLC cells were injected into mice via the tail vein. Lung metastasis was enhanced in KO mice compared to WT mice, as illustrated by bioluminescence imaging (Fig. 1e, f). Noticeably, histological examination revealed that compared with WT mice, KO mice had larger and more lung metastasis nodules with increased Ki67 staining (Fig. 1g–j and Supplementary Fig. 1k-m), leading to shortened overall survival for the KO mice compared with the WT mice (Fig. 1k and Supplementary Fig. 1n). In addition, we observed that the expression of METTL3 was attenuated in TAMs compared to macrophages in normal tissue (Fig. 1l).

**Fig. 2:**
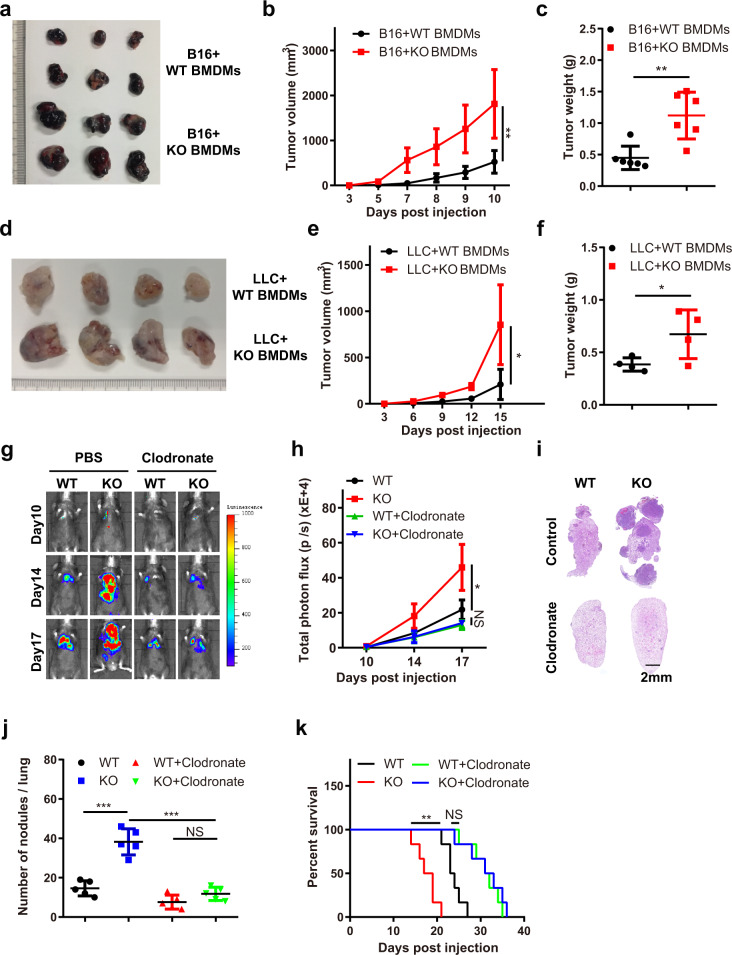
Macrophages with *Mettl3* depletion enhance tumour growth and lung metastasis. **a**–**c** WT mice were subcutaneously inoculated with 1 × 10^6^ LLC cells mixed with 2.5 × 10^5^ WT or KO macrophages. Tumours were dissected 10 days after inoculation and photographed (**a**). Tumour volume (**b**) and tumour weight (**c**) were recorded (*n* = 6 mice per group). **d**–**f** WT mice were subcutaneously inoculated with 1 × 10^6^ B16 cells mixed with 2.5 × 10^5^ WT or KO macrophages. Tumours were dissected 15 days after inoculation and photographed (**d**). Tumour volume (**e**) and tumour weights (**f**) were recorded (*n* = 4 mice per group). **g**–**k** WT and KO mice were injected with clodronate liposomes 2 days before experiments. Then, B16 cells were intravenously injected into the mice. Bioluminescence was measured at the indicated time points, and representative images are shown (**g**) and were quantified (**h**) (*n* = 3 mice per group). Representative HE staining images of lung sections are shown (**i**). Lung metastatic nodules (**j**) (*n* = 5 mice per group) and overall survival (**k**) (*n* = 6 mice per group) were observed. Data are shown as the mean ± SD. *P* values were determined by a two-tailed *t*-test (**b**, **c**, **e**, **f**, **h** and **j**) and the Gehan–Breslow–Wilcoxon test (**k**). *P ≤ 0.05, **P < 0.01 and ***P < 0.001. NS (non-significant) means *P* > 0.05. The source data are provided as a Source data file.

To explore whether the loss of *Mettl3* in BMDMs enhances tumour growth, we established a mixed model of macrophages and tumour cells. BMDMs were labelled with CFSE and co-injected with B16 or LLC cells into mice subcutaneously. Then, the tumours were extracted, and immunofluorescence staining for F4/80 was performed. The results showed that the CFSE-labelled cells were positive for F4/80 staining, which indicated that the co-injected BMDMs could survive for 2 weeks in tumours (Supplementary Fig. 2a). The extracellular matrix in tumour was also determined by COL1A1 staining (Supplementary Fig. 2b). In addition, we observed that the co-injected BMDMs had almost no proliferative capacity (Supplementary Fig. 2c). Further analysis showed that co-injection of B16 or LLC cells with KO BMDMs resulted in significantly accelerated tumour growth (Fig. 2a–f and Supplementary Fig. 2d). Next, we depleted macrophages in mice by intravenous treatment with clodronate liposomes two days before experiments. Then, B16 cells were intravenously injected into the mice. Bioluminescence imaging revealed that the differences in tumour growth and metastasis between WT and KO mice were abrogated by macrophage depletion (Fig. 2g, h), which was confirmed by histological analysis of lung tumours (Fig. 2i, j). The survival of the depleted mice was extended, with no remarkable difference between WT and KO mice treated with clodronate liposomes (Fig. 2k). Taken together, the data above suggested a vital role for METTL3 in macrophages in promoting tumour progression.

### Mettl3 depletion in macrophages reshapes the tumour microenvironment by enhancing M1- and M2-like TAM and Treg infiltration into tumours

**Fig. 3:**
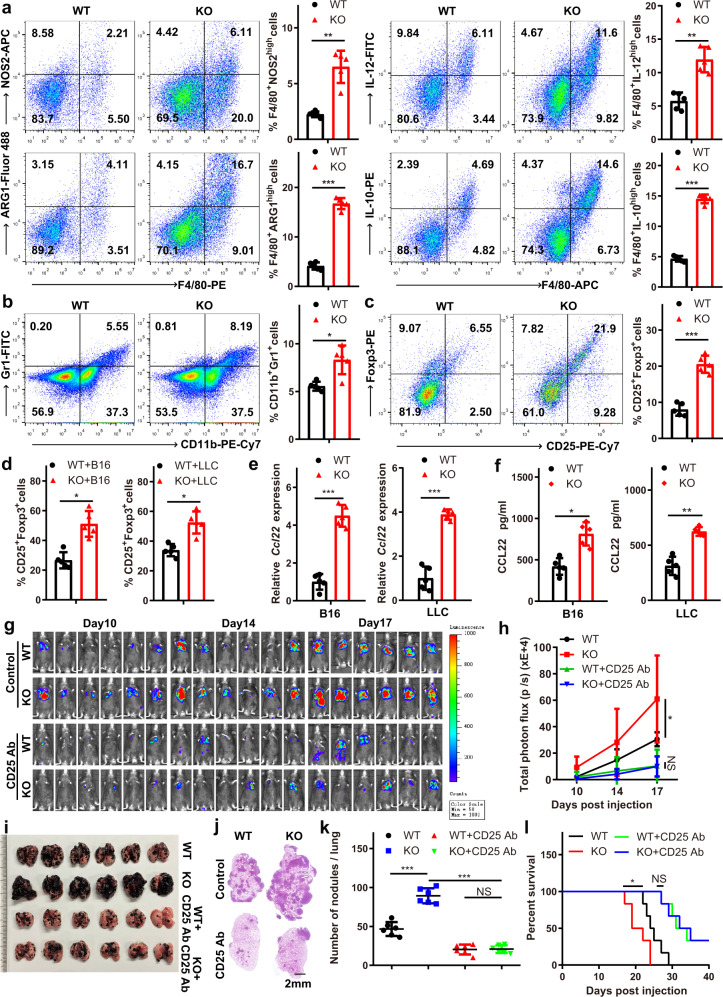
*Mettl3* depletion in macrophages establishes an immunosuppressive microenvironment by enhancing M1- and M2-like TAM and Treg infiltration in tumours. **a** Flow cytometry analysis of macrophage subpopulations in B16 tumours from tumour-bearing mice. The percentages of M1-like (F4/80^+^IL-12^high^ or F4/80^+^NOS2^high^) and M2-like (F4/80^+^IL-10^high^ or F4/80^+^ARG1^high^) TAMs were calculated, with CD11b^+^ cells gated (*n* = 5 mice per group). **b** Single-cell suspensions of B16 tumours were stained with fluorophore-conjugated antibodies specific for CD11b and Gr1 and analysed by flow cytometry (*n* = 5 mice per group). **c** B16 tumours were dissected and digested to obtain single-cell suspensions. Infiltrating Treg (CD25^+^Foxp3^+^) cells were stained with fluorophore-conjugated antibodies and analysed by flow cytometry, with CD4^+^ cells gated (*n* = 5 mice per group). **d** BMDMs were subcutaneously co-injected with B16 or LLC cells into mice. Treg (CD25^+^Foxp3^+^) cell infiltration was analysed by flow cytometry, with CD4^+^ cells gated (*n* = 5 mice per group). **e** qRT-PCR analysis of the expression of *Ccl22* in WT and KO TAMs from B16 or LLC tumours (*n* = 5 mice per group). **f** ELISA analysis of CCL22 in WT and KO TAMs from B16 or LLC tumours (*n* = 5 mice per group). **g**–**l** B16 cells were intravenously injected into WT and KO mice. An anti-CD25 Ab was injected on days −2, 1, 4, 7 and 11. Bioluminescence was measured at the indicated time points, and representative images are shown (**g**) and were quantified (**h**) (*n* = 6 mice per group). Lungs were dissected and photographed (**i**), and representative HE staining images of lung sections are shown (**j**) (*n* = 6 mice per group). Lung metastatic nodules (**k**) and overall survival (**l**) were observed (*n* = 6 mice per group). Data are shown as the mean ± SD. *P* values were determined by a two-tailed *t*-test (**a**–**f, h** and **k**) and the Gehan–Breslow–Wilcoxon test (**l**). *P ≤ 0.05, **P < 0.01 and ***P < 0.001. NS (non-significant) means *P* > 0.05. The source data are provided as a Source data file.

To assess the effects of METTL3 on the immune microenvironment, we performed immunophenotyping of the spleen and lungs by flow cytometry and determined that the overall percentages of M1 macrophages (CD11b^+^F4/80^+^NOS2^high^ or CD11b^+^F4/80^+^IL-12^high^), M2 macrophages (CD11b^+^F4/80^+^ARG1^high^ or CD11b^+^F4/80^+^IL-10^high^) and regulatory T cells (CD4^+^CD25^+^Foxp3^+^) were increased (Supplementary Figs. 3a–c and 4a–c), while the overall percentages of MDSCs (CD11b^+^Gr1^+^) and CD3^+^CD4^+^ and CD3^+^CD8^+^ T cells were not affected in KO mice (Supplementary Figs. 3d, e and 4d, e). To further investigate the impact of METTL3 on the tumour microenvironment, we analysed CD45+ cells in tumours from WT or KO mice through single-cell RNA sequencing. Further analysis of the data revealed 23 distinct intratumoural immune cell populations. Specifically, we identified macrophages (7 cell clusters), MDSCs (2 cell clusters), granulocytes (2 cell clusters), CD4^+^ T cells (1 cell cluster), CD8^+^ T cells (2 cell clusters), natural killer (NK) cells (1 cell cluster), B cells (2 cell clusters), dendritic cells (DCs, 2 cell clusters), plasmacytoid dendritic cells (pDCs, 1 cell cluster), conventional dendritic cells (cDCs, 1 cell cluster), fibroblasts (1 cell cluster) and tumour cells (1 cell cluster) (Supplementary Fig. 5a). The tumours from KO mice showed differences in the composition of the immune infiltrate compared with the tumours from WT mice, which included increases in the numbers of TAMs, MDSCs and CD4^+^ T cells and trends towards decreased numbers of granulocytes and CD8^+^ T cells (Supplementary Fig. 5b). Moreover, immunophenotyping of tumour tissues with METTL3-deficient macrophages through flow cytometry showed an increase in the overall percentage of M1- and M2-like TAMs (Fig. 3a and Supplementary Fig. 6a). In addition, there were trend towards increases in the numbers of MDSCs and regulatory T cells (Fig. 3b–d and Supplementary Figs. 3c, 6b), whereas the overall percentages of CD3^+^CD4^+^ and CD3^+^CD8^+^ T cells were not affected (Supplementary Figs. 3e and 6c, d). Immunohistochemical staining of tumour sections also produced similar results (Supplementary Fig. 6e-g). Previous studies have shown that Treg migration and infiltration into various tumour tissues appear to be dependent on the expression of the CCR4 ligand (CCL22) produced by tumour cells or infiltrating macrophages^3,29^. Further analysis showed that CCL22 expression was remarkably upregulated in *Mettl3*-deficient BMDMs and TAMs, as measured by qRT-PCR and ELISA (Fig. 3e, f and Supplementary Fig. 6h). These findings prompted us to examine the impacts of Treg depletion on tumour growth and metastasis. We first investigated the effect of injecting anti-CD25 antibodies on the level of Tregs. As shown in Supplementary Fig. 7a, an ~75% reduction in the Treg level was observed. It was also noted that with the reduction in the Treg cell population, the percentages of Th1 cells (CD4^+^IFN-γ^+^) and IFN-γ^+^ CD8 T cells were increased in tumours, while that of Th2 cells (CD4^+^IL-4^+^) was almost unaffected (Supplementary Fig. 7b-d). Further analysis showed that WT and KO mice that received anti-CD25 antibodies for Treg depletion displayed no significant differences in tumour growth or metastasis, while increased tumour growth was observed in KO mice compared with WT mice in the untreated group (Fig. 3g–k). The difference in survival between WT and KO mice was also abolished upon Treg depletion (Fig. 3l). These data suggested that *Mettl3* depletion in myeloid cells promoted tumour growth and metastasis in a manner dependent on M1/M2-like TAM infiltration and Treg recruitment.

### Mettl3 depletion facilitates the M1 and M2 polarization of BMDMs through NF-kB/STAT3

**Fig. 4:**
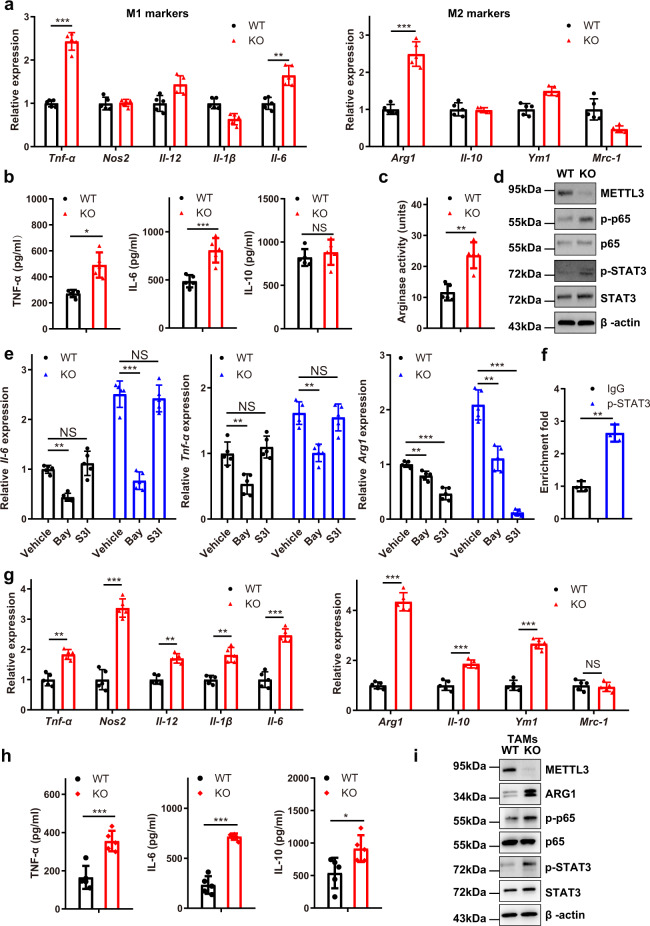
*Mettl3* depletion facilitates the M1 and M2 polarization of BMDMs through NF-kB/STAT3. **a** qRT-PCR analysis of M1 and M2 macrophage-associated gene expression in WT and KO BMDMs (*n* = 5 mice per group). **b** ELISA analysis of TNF-α, IL-6 and IL-10 secretion by WT and KO BMDMs (*n* = 5 mice per group). **c** Arginase activity in WT and KO BMDMs (*n* = 5 mice per group). **d** Western blot analysis of p65, p-p65, STAT3 and p-STAT3 in WT and KO BMDMs. The blots are representative of *n* = 3 independent experiments. **e** qRT-PCR analysis of *Tnf-α*, *Il-6* and *Arg1* in WT and KO BMDMs treated with the NF-κB inhibitor BAY-11-7082 (5 µM) or STAT3 inhibitor S3I-201 (100 µM) for 8 h (*n* = 5 mice per group). **f** A ChIP assay was performed with an anti-p-STAT3 antibody, followed by PCR amplification of the *Arg1* promoter. *n* = 3 independent experiments. **g** WT and KO TAMs were isolated from tumour-bearing mice. M1 and M2 macrophage-associated gene expression was assessed by qRT-PCR (*n* = 5 mice per group). **h** The expression of TNF-α, IL-6 and IL-10 in TAMs isolated from B16 tumour-bearing mice was analysed by ELISA (*n* = 5 mice per group). **i** The expression of ARG1, p65, p-p65, STAT3 and p-STAT3 in TAMs was determined by immunoblotting. The blots are representative of *n* = 3 independent experiments. Data are shown as the mean ± SD. *P* values were determined by a two-tailed *t*-test (**a**–**c** and **e**–**h**). *P ≤ 0.05, **P < 0.01 and ***P < 0.001. NS (non-significant) means *P* > 0.05. The source data are provided as a Source data file.

To characterize the potential involvement of METTL3 in macrophage polarization, BMDMs were treated with LPS and IFN-γ to induce M1 macrophages or with IL-4 to induce M2 macrophages. qRT-PCR results showed that the expression of *Tnf-α*, *Il-6* and *Arg1* was noticeably upregulated in *Mettl3*-deficient BMDMs (Supplementary Fig. 8a, b). Next, we tested the expression of M1 and M2 macrophage-associated genes and found that the expression of *Tnf-α*, *Il-6* and *Arg1* was significantly increased in *Mettl3*-deficient BMDMs, consistent with previous results (Fig. 4a). ELISA also revealed that the expression of TNF-α and IL-6 was upregulated in *Mettl3*-deficient BMDMs, while that of IL-10 was relatively unchanged (Fig. 4b). Increased arginase activity was also observed in *Mettl3*-deficient BMDMs (Fig. 4c). NF-κB is a central player in host innate and adaptive immune responses that regulates the expression of pro-inflammatory cytokines^30^, and STAT3 is one of the major drivers of MDSC expansion and is involved in myeloid cell-mediated immune suppression^31^. We evaluated p65 and STAT3 phosphorylation in BMDMs and observed increased levels of p65 and STAT3 phosphorylation in *Mettl3*-deficient macrophages (Fig. 4d). Inhibition of NF-κB reduced the expression of *Tnf-α*, *Il-6* and *Arg1*, while inhibition of the STAT3 pathway reduced the expression of *Arg1* in both WT BMDMs and KO BMDMs (Fig. 4e). ChIP analysis demonstrated that p-STAT3 specifically bound to the promoter of *Arg1* (Fig. 4f). We next harvested TAMs from tumour-bearing mice and the purity of TAMs tested by flow cytometry was shown in Supplementary Fig. 9. The results showed that the expression of M1 and M2 macrophage-associated genes in KO TAMs was increased simultaneously, which was associated with the enhanced phosphorylation of p65 and STAT3 and expression of ARG1 (Fig. 4g–i). Collectively, these data suggested that the loss of METTL3 resulted in activation of the NF-κB pathway and STAT3 signalling, which led to M1- and M2-like macrophage polarization.

### Tumour cells enhance the production of cytokines and activation of NF-κB and STAT3 in Mettl3-depleted macrophages

As NF-κB and STAT3 can be regulated by extracellular stimuli, we stimulated BMDMs with the exogenous factor TNF-α or IL-6. Immunoblot results revealed that the capacity to induce p-p65 and p-STAT3 was more apparent in KO BMDMs than in WT BMDMs (Supplementary Fig. 10a, b). qRT-PCR analysis showed that the expression of *Tnf-α* and *Il-6* increased in response to TNF-α treatment, which was reversed by blocking the NF-κB pathway with Bay-11-7082, while the expression of *Arg1* increased in response to IL-6 treatment, which was reversed by the STAT3 inhibitor S3I-201 (Supplementary Fig. 10c–e). These observations implied that the NF-κB/IL-6/STAT3 signalling axis was more activated in KO BMDMs than in WT BMDMs.

**Fig. 5:**
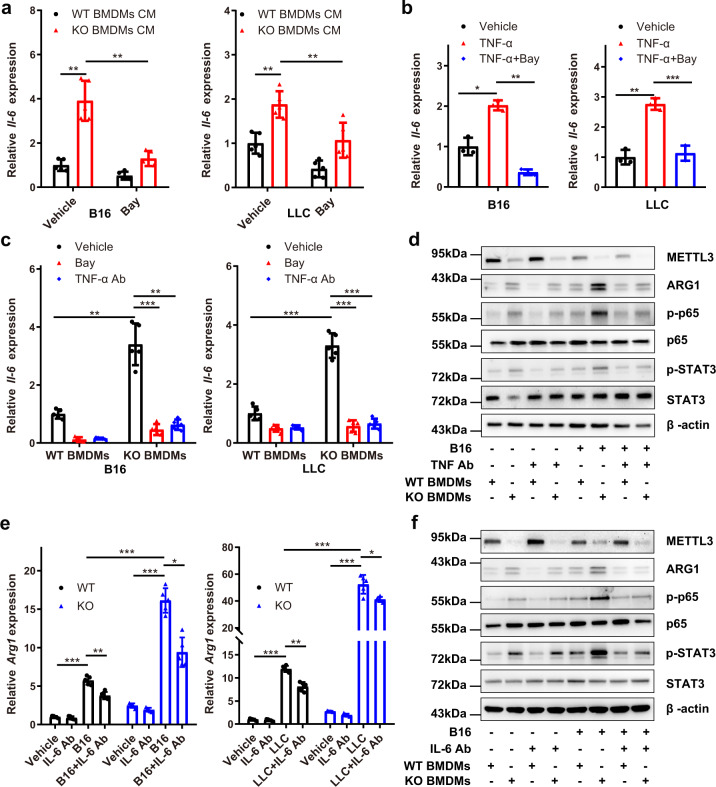
Production of cytokines and activation of NF-κB and STAT3 are enhanced in *Mettl3*-null macrophages co-cultured with tumour cells. **a** qRT-PCR analysis of *Il-6* expression levels in B16 and LLC cells incubated with WT or KO BMDM culture medium for 24 hours in the presence or absence of BAY-11-7082 (5 µM) (*n* = 5 mice per group). **b** B16 and LLC cells were treated with TNF-α (40 ng/mL) or TNF-α + BAY-11-7082 (5 µM), followed by qRT-PCR analysis of *Il-6*. *n* = 3 independent experiments. **c** B16 and LLC cells co-cultured with WT or KO BMDMs in the presence of BAY-11-7082 (5 µM) or an anti-TNF-α Ab (20 µg/mL) for 8 h. qRT-PCR analysis of *Il-6* expression levels in WT or KO BMDMs was performed (*n* = 5 mice per group). **d** Western blot analysis of the indicated proteins in WT and KO BMDMs co-cultured with B16 cells in the presence or absence of the anti-TNF-α Ab (20 µg/mL). The blots are representative of *n* = 3 independent experiments. **e** qRT-PCR analysis of *Il-6* expression in WT and KO BMDMs co-cultured with B16 or LLC cells in the presence or absence of an anti-IL-6 Ab (20 µg/mL) (*n* = 5 mice per group). **f** Western blot analysis of the indicated proteins in WT and KO BMDMs co-cultured with B16 cells in the presence or absence of the anti-IL-6 Ab (20 µg/mL). The blots are representative of *n* = 3 independent experiments. Data are shown as the mean ± SD. *P* values were determined by a two-tailed *t*-test (**a**–**c** and **e**). *P ≤ 0.05, **P < 0.01 and ***P < 0.001. The source data are provided as a Source data file.

To assess the role of the NF-κB/IL-6/STAT3 signalling axis in the tumour microenvironment, B16 or LLC cells were incubated with BMDM culture medium or BMDMs. The results showed that the expression of *Il-6* was upregulated in B16 and LLC cells with increased p65 phosphorylation (Supplementary Fig. 10f-h) and reversed by blocking the NF-κB pathway with Bay-11-7082 (Fig. 5a). We further found that TNF-α treatment increased not only the phosphorylation of p65 but also the expression of *Il-6* in a dose- and time-dependent manner (Supplementary Fig. 10i, j) and that treatment with the NF-κB inhibitor BAY-11-7082 reversed the expression of *Il-6* in tumour cells (Fig. 5b). Furthermore, WT or KO BMDMs were pre-incubated with Bay-11-7082 or anti-TNF-α neutralizing antibodies and then co-cultured with B16 or LLC cells. qRT-PCR analysis showed that the expression of *Il-6* was inhibited by the addition of Bay-11-7082 or the anti-TNF-α neutralizing antibodies (Fig. 5c), accompanied by reduced p65 and STAT3 phosphorylation (Fig. 5d). Furthermore, the expression of *Arg1* was significantly decreased in *Mettl3*-deficient BMDMs co-cultured with B16 or LLC cells in the presence of anti-IL-6 neutralizing antibodies compared with WT BMDMs (Fig. 5e, f). In summary, these data suggested that *Mettl3*-deficient macrophages reshaped the tumour microenvironment by regulating cytokine responses.

### Loss of METTL3 impairs the YTHDF1-mediated translation of SPRED2

**Fig. 6:**
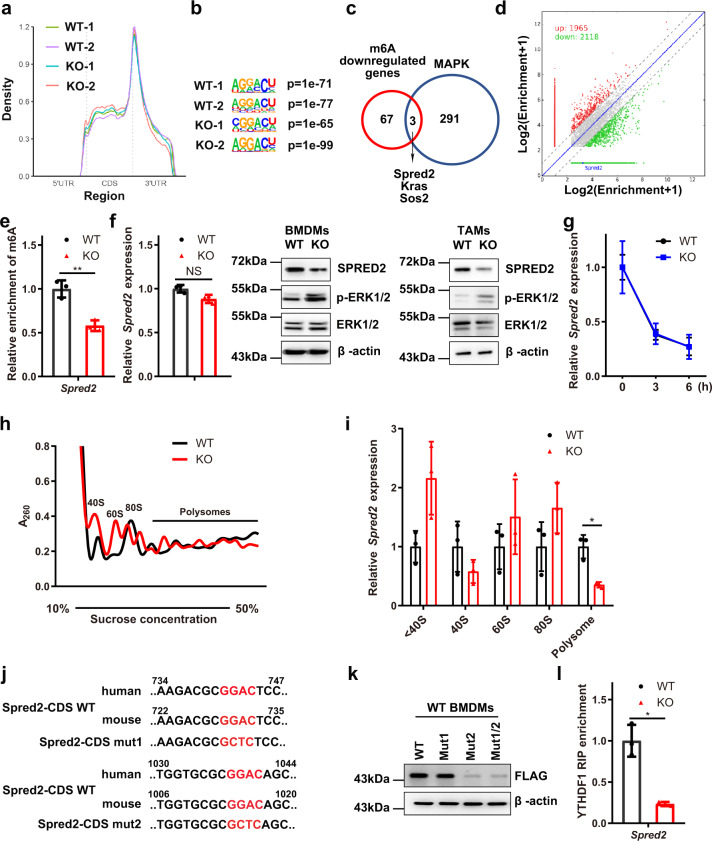
Loss of METTL3 impairs the YTHDF1-mediated translation of SPRED2. **a** Metagene profiles of the m6A distribution across the transcriptome in WT and KO BMDMs. **b** Consensus sequence motif for m6A methylation identified in WT and KO BMDMs. **c** Overlap between m6A-downregulated genes (fold-change >4 and *P* value < 0.00001) in BMDMs and MAPK pathway-related functional genes. **d** Scatter plots showing m6A-related changes in cellular transcript levels in KO BMDMs versus WT BMDMs. **e** m6A enrichment in *Spred2* mRNA in WT and KO BMDMs, as determined by m6A-RIP-qPCR. *n* = 3 independent experiments. **f** qRT-PCR (left panel) of SPRED2 expression in WT and KO BMDMs (*n* = 3 mice per group). The expression of SPRED2 in BMDMs (middle panel) and TAMs (right panel) was determined by immunoblotting. The blots are representative of *n* = 3 independent experiments. **g** *Spred2* mRNA stability in WT and KO BMDMs treated with actinomycin D at the indicated times determined by qRT-PCR (*n* = 3 mice per group). **h** A representative polysome gradient profile for WT and KO BMDMs. **i** Analysis of *Spred2* mRNA in the non-ribosomal fraction (<40S), 40S, 60S, 80S and polysomes of KO BMDMs compared to those of control cells. *n* = 3 independent experiments. **j** Schematic representation of mutations in the conserved coding sequence (CDS) for the investigation of the roles of m6A in SPRED2 expression. **k** FLAG-SPRED2-CDS WT, FLAG-SPRED2-CDS mut1, FLAG-SPRED2-CDS mut2 or FLAG-SPRED2-CDS mut1/2 was transfected into BMDMs for 24 h. Protein expression was measured by western blotting. The blots are representative of *n* = 3 independent experiments. **l** RNA immunoprecipitation (RIP) analysis of the interaction of *Spred2* mRNA transcripts with YTHDF1 in WT and KO BMDMs. Enrichment of *Spred2* was measured by qRT-PCR. *n* = 3 independent experiments. Data are means ± SD. *P* values were determined by hypergeometric test (**b**) and two-tailed *t*-test (**c**, **e**–**g**, **i** and **l**). *P ≤ 0.05 and **P < 0.01. NS (non-significant) means *P* > 0.05. The source data are provided as a Source data file.

To investigate the roles of m6A in macrophage reprogramming, total RNA samples were isolated from WT and KO BMDMs for m6A profiling using m6A-methylated RNA immunoprecipitation sequencing (MeRIP-Seq). The density of m6A peaks increased steadily along the transcript in the CDS and decreased in abundance along the length of the 3′-UTR (Fig. 6a). Motif searching identified the consensus “GGAC” motif within the m6A sites (Fig. 6b). To characterize potential targets involved in m6A-regulated macrophage polarization, we identified candidate genes with overlap between MAPK pathway-related genes (listed in Supplementary Table 1) with key functions and 70 m6A-downregulated genes. Among these genes, *Spred2*, which was found to have significantly decreased m6A levels (Fig. 6c, d), has been suggested to be a negative regulator controlling ERK signalling^32^. Furthermore, m6A immunoprecipitation (m6A-IP) followed by qRT-PCR confirmed that *Spred2* was an m6A-regulated target gene (Fig. 6e). We next determined that SPRED2 protein expression was decreased in KO BMDMs and TAMs, while the mRNA level was not noticeably altered (Fig. 6f). mRNA decay assays demonstrated that m6A modification did not appreciably affect the decay of *Spred2* (Fig. 6g). We hypothesized that the downregulation of SPRED2 protein expression in *Mettl3*-deficient BMDMs may be due to a difference in protein translation efficiency controlled by YTHDF1, the m6A reader protein that promotes translation of m6A-methylated transcripts^33^. Next, we separated the isolated RNA into the non-translating fraction (<40S), translation initiation fraction (including 40S ribosomes, 60S ribosomes and 80S monosomes) and translation-active polysomes (>80S) from WT and KO BMDMs by polysome profiling (Fig. 6h). qRT-PCR showed that the *Spred2* mRNA level in translation-active polysomes (>80S) of *Mettl3*-deficient BMDMs was significantly lower than that in WT BMDMs (Fig. 6i). Recent studies have shown that promoter-bound METTL3 augments the translation of its target genes^18,34^. We next tested whether METTL3 is recruited to the *Spred2* promoter and affects *Spred2* expression. For this, we used a reporter system consisting of a plasmid harbouring *Spred2* promoter fragments upstream of the firefly luciferase gene. The results showed that neither wild-type METTL3 (WT METTL3) nor a catalytically dead mutant of METTL3 (mut METTL3; residues 395–398: DPPW → APPA) could influence the luciferase activity (Supplementary Fig. 11a). To directly address the requirement for the catalytic activity of METTL3 for the translation of SPRED2, we overexpressed WT METTL3 or mut METTL3 in BMDMs. The results demonstrated that overexpression of WT METTL3 but not overexpression of mut METTL3 promoted the translation of SPRED2 (Supplementary Fig. 11b). The results indicated that the catalytic activity of METTL3 played a vital role in SPRED2 translation independent of promoter binding. To investigate whether m6A methylation in the CDS can regulate the expression of SPRED2, we analysed the conserved m6A motifs of *Spred2* in humans and mice and mutated the two potentially conserved m6A motifs GGAC to GCTC independently (*Spred2*-CDS mut1 or *Spred2*-CDS mut2) or simultaneously (*Spred2*-CDS mut1/2). Our data showed that the SPRED2 level was reduced with *Spred2*-CDS mut2 and *Spred2*-CDS mut1/2 but not with *Spred2*-CDS mut1, which indicated that the m6A motif in *Spred2*-CDS mut2 was the main site for expression regulation (Fig. 6j, k). Furthermore, RNA-IP analysis indicated that the binding between YTHDF1 and *Spred2* was significantly decreased in *Mettl3*-deficient BMDMs compared with WT BMDMs (Fig. 6l).

To confirm the roles of YTHDF1 in m6A-regulated SPRED2 expression, we knocked down the expression of YTHDF1 in BMDMs. The results showed that YTHDF1 knockdown attenuated the expression of SPRED2 in BMDMs, accompanied by increased ERK, NF-κB and STAT3 phosphorylation (Supplementary Fig. 12a). qRT-PCR analysis showed that the expression of *Tnf-α*, *Il-6*, *Arg1* and *Ccl22* increased upon YTHDF1 knockdown (Supplementary Fig. 12b-e). Furthermore, SPRED2 knockdown in BMDMs resulted in upregulation of ERK, NF-κB and STAT3 phosphorylation (Supplementary Fig. 12f), accompanied by increased expression of *Tnf-α*, *Il-6*, *Arg1* and *Ccl22* (Supplementary Fig. 12g-j). Overall, we found that loss of *Mettl3* in BMDMs led to a reduction in the YTHDF1-mediated translation of SPRED2 and upregulation of ERK, NF-κB and STAT3 phosphorylation.

### Mettl3 depletion in myeloid cells impairs PD-1 blockade therapeutic efficacy in B16 melanoma

**Fig. 7:**
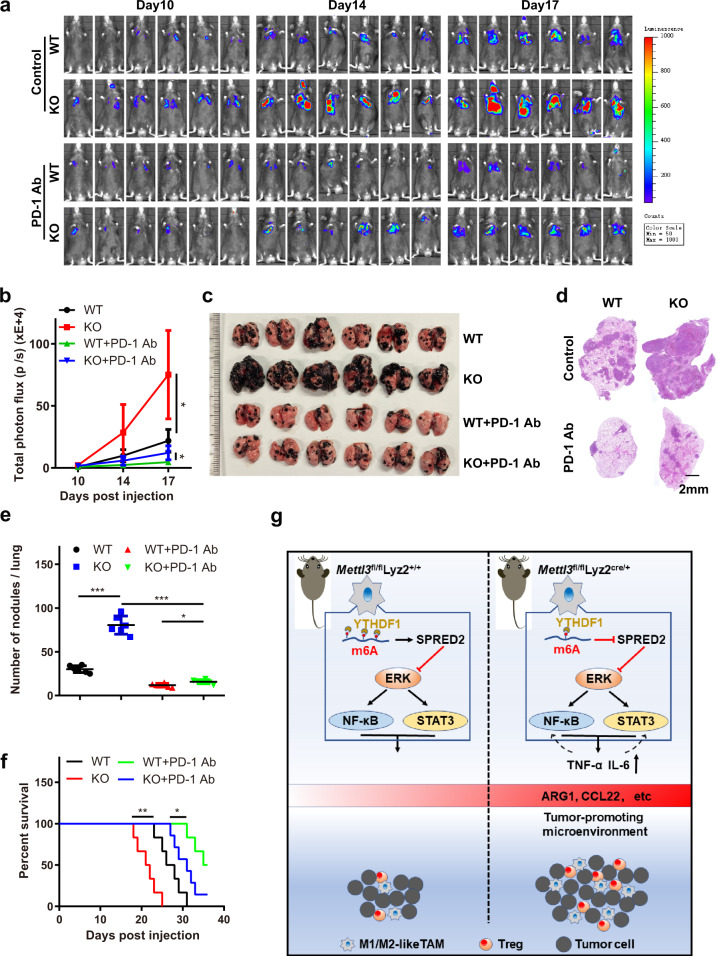
*Mettl3* depletion in macrophages impairs PD-1 blockade therapeutic efficacy in B16 tumours. **a**–**f** B16 cells were intravenously injected into WT and KO mice. An anti-PD-1 Ab was injected on days 0, 3, 6, 9, 12. Bioluminescence was measured at the indicated time points, and representative images are shown (**a**) and were quantified (**b**) (*n* = 6 mice per group). Lungs were dissected and photographed (**c**), and representative HE staining images of lung sections are shown (**d**). Lung metastatic nodules (**e**) (*n* = 6 mice per group) and overall survival (**f**) were observed (WT, KO or WT + PD-1 Ab group, *n* = 6 mice; KO + PD-1 Ab group, *n* = 7 mice). **g** Schematic showing that *Mettl3* ablation in myeloid cells impairs the translation of SPRED2 mediated by YTHDF1, which facilitates tumour progression by regulating cytokine responses and enhancing tumour-promoting macrophage and Treg infiltration. Data are shown as the mean ± SD. *P* values were determined by a two-tailed *t*-test (**b**, **e**) and the Gehan–Breslow–Wilcoxon test (**f**). *P ≤ 0.05, **P < 0.01 and ***P < 0.001. The source data are provided as a Source data file.

To evaluate the potential of targeting METTL3 to activate antitumour responses, we tested anti-PD-1 therapy in B16 tumour metastasis models. As expected, we found that B16 tumours were less responsive to anti-PD-1 therapy in KO mice, which exhibited greatly increased tumour growth and lung metastasis and shortened survival (Fig. 7a–f). This implied that *Mettl3*-deficient myeloid cells contributed to B16 tumour resistance to anti-PD-1 therapy.

## Discussion

Macrophages were the first “immune” cells to appear in evolution and are present in virtually all tissues^35^. However, macrophages are also an enigma. To date, the macrophage field has arrived at a partial consensus on the description of the broad grouping of macrophage activation phenotypes^36–38^. However, the use of the terms M1 and M2 remains controversial because of the lack of tightly defined criteria to score phenotypes. In our study, we found that the expression of M1-associated genes (*Tnf-α* and *Il-6*) and M2-associated genes (*Arg1*) was significantly increased in *Mettl3*-deficient BMDMs, accompanied by increased NF-κB and STAT3 phosphorylation. Our data are consistent with a previous report demonstrating that METTL3 significantly attenuates the LPS-induced inflammatory response in macrophages^39^ but seemingly contradict another study showing that METTL3 facilitates M1 macrophage polarization^40^. In addition, we identified *Spred2* as an m6A target gene. METTL3 depletion attenuated the translation of SPRED2 mediated by YTHDF1. Previous studies have shown that *Spred2*-knockout mice have more infiltrating macrophages with an M1 phenotype in mesenteric white adipose tissue^41^ and produce significantly higher levels of inflammatory cytokines^42^, which seems to partly differ from our results. The results also indicate that the dynamic plasticity of macrophages is regulated by the local microenvironment, which makes it possible to target macrophages for disease therapy. Inflammatory cytokines are part of the immune response that either increase or decrease inflammation. TNF is important in innate immunity, inflammation and host defence against microbial pathogens^43^. Classical inflammatory activation of cells by TNF is mediated by canonical NF-κB signalling. TNF also has potent paradoxical anti-inflammatory functions that limit inflammation-associated toxicity^44^. IL-6 has dual functions in the immune system: it exerts a pro-inflammatory effect^45^ or an anti-inflammatory^46^ effect depending on the local immune microenvironment. Recent evidence suggests that IL-6 is a central player linking chronic inflammation to cancer by driving tumour initiation and subsequent growth and metastasis^47^. Our work demonstrated that METTL3-dependent signalling led to activation of NF-κB and STAT3, which in turn caused autocrine TNF-α/IL-6-induced activation of NF-κB and STAT3. Moreover, TNF-α derived from TAMs could stimulate tumour cells to release IL-6, contributing to STAT3 activation in TAMs.

Specific subsets of immune cells have been identified as key drivers of neoplastic progression, such as TAMs, MDSCs and Tregs^47^. Different lymphocytes traffic into the tumour microenvironment and modulate antitumour immune responses. Our results demonstrated that the percentages of M1/M2-like TAMs and Tregs were significantly increased in tumours from KO mice, which might establish a beneficial context for tumour growth and metastasis. In the tumour microenvironment, chemokines can be expressed by tumour cells and other cells to recruit different subsets of immune cells. We found that *Mettl3*-deficient TAMs expressing relatively high levels of CCL22 were responsible for Treg recruitment within the complex tumour microenvironment. Treg depletion abrogated the enhanced tumour growth and metastasis observed in *Mettl3*-deficient mice. Numerous studies have shown that TAMs suppress T-cell function. This was often thought to relate to ARG1 expression by TAMs leading to more metabolism of l-arginine, which is necessary for T-cell fitness and antitumour activity^48,49^. Genetic ablation of *Arg1* in the myeloid compartment of tumour-bearing mice was shown to reduce tumour growth, indicative of an immunosuppressive role for ARG1 in vivo^50,51^. Our results showed that the expression of ARG1 was markedly upregulated in *Mettl3*-deficient myeloid cells, enhancing tumour growth and metastasis. In addition, we found that the percentages of Th1 cells (CD4^+^IFN-γ^+^) and IFN-γ^+^CD8^+^ cells were reduced in tumours from KO mice. Together, these data demonstrated that METTL3 deficiency in macrophages contributed to the formation of an immunosuppressive microenvironment, including increased Treg infiltration and reduced Th1 cells and IFN-γ^+^CD8^+^ cells, and subsequently facilitated tumorigenesis.

Lyz2-cre has been extensively applied to characterize the systemic functions of endogenous macrophages. However, in addition to affecting monocytes and mature macrophages, Lyz2-cre also caused METTL3 depletion in most granulocytes and a small number of CD11c^+^ dendritic cells (DCs) in mice^52^. ScRNA-seq results revealed that fewer granulocytes infiltrated into tumours from KO mice than those from WT mice and the infiltration of DCs did not seem to be affected. Therefore, the involvement of granulocytes and DCs in the tumour microenvironment still needs to be further explored in the future. In summary, our findings reveal a non-cell-intrinsic, tumour-suppressing function for METTL3, as ablation of METTL3 in macrophages leads to tumour expansion and metastasis. Our work support further exploring the m6A regulatory pathway in macrophages to clarify the immune regulation of solid tumours. However, we could not exclude the effect of chromosome-associated regulatory RNAs (carRNAs) methylation mediated by METTL3^53^. In addition, acute inhibition of METTL3 in adult could be different comparing to studies of KO mice here. Further mechanistic studies in this area may provide additional insights into the m6A regulatory pathway in macrophages for clinical intervention.

## Methods

### Mice

Mice with floxed *Mettl3* were generated by Beijing Biocytogen Co., Ltd. Lyz2-Cre mice (catalogue 004781, The Jackson Laboratory) were crossed with floxed *Mettl3* mice to generate *Mettl3*^fl/fl^Lyz2^+/+^ (WT) and *Mettl3*^fl/fl^Lyz2^cre/+^ (KO) mice. All experimental mice were bred and maintained under specific pathogen-free conditions, fed standard laboratory chow, and kept on a 12-h light/dark cycle and temperature and humidity were kept at 22 ± 1 °C, 55% ± 5%. All mice were on the C57BL/6 genetic background, maintained in individual cages and used between 6 and 12 weeks of age. Co-housed Cre-negative littermate mice were used as control animals in all experiments. All animal experiments were approved by the Animal Experiment Administration Committee of Fourth Military Medical University.

### Cell culture

The mouse cell lines B16 and LLC were obtained from the American Type Cell Culture Collection (ATCC). All cell lines were cultured in Dulbecco’s modified Eagle’s medium (DMEM, Gibco) supplemented with 10% foetal bovine serum (FBS, Gibco). Cells were incubated at 37 °C with 5% CO_2_.

### Inhibitors and transfection

The NF-κB inhibitor BAY-11-7082 and STAT3 inhibitor S3I-201 were obtained from Selleck. For siRNA transfection, cells were seeded one day before transfection. Transfection was performed at ~60% confluence using Lipofectamine RNAiMAX (Thermo Fisher Scientific) according to the manufacturer’s instructions. The siRNA duplex oligonucleotides used are listed in Supplementary Table 2.

### Bone marrow-derived macrophage (BMDM) differentiation and polarization

The murine bone marrow-derived macrophages (BMDMs) used in morphological and transcriptional assays to assess polarization were isolated and derived as previously described^54^ with some modifications. Briefly, bone marrow was extracted from the femurs and tibias of C57BL/6 mice, and red blood cells were lysed. The resultant bone marrow cells were cultured in Dulbecco’s modified Eagle’s medium (DMEM) supplemented with 10% FBS, 100 U/mL penicillin and 100 mg/mL streptomycin in the presence of 40 ng/mL M-CSF for 7 days prior to treatment. BMDMs were stimulated with 50 ng/ml LPS and 40 ng/ml IFN-γ or 40 ng/ml IL-4 (PeproTech) separately for 24 h to obtain M1 or M2 macrophages, respectively. Conditioned medium from B16 or LLC cells was used to stimulate BMDMs to generate TAMs in vitro.

### Flow cytometry and cell sorting

Tissues or tumours were excised from host mice, minced, digested with 10 U/mL collagenase I (Gibco) and 30 U/mL DNase I in RPMI medium for 60 min at 37 °C, and filtered through a 40-μm nylon filter (BD Biosciences) to obtain single-cell suspensions. After the red blood cells were lysed, the remaining cells were washed twice with complete RPMI medium and stained with antibodies specific for surface markers. The following Abs were purchased from BD Biosciences: anti-CD11b-PE-Cy7 (1:100, M1/70), anti-Gr1-FITC (1:100, RB6-8C5), anti-CD25-PE-Cy7 (1:100, PC61), anti-F4/80-APC (1:100, T45-2342), anti-F4/80-PE (1:100, T45-2342), anti-CD3-FITC (1:100, 17A2), anti-CD4-APC (1:100, RM4-5), anti-CD4-FITC (1:100, RM4-5), and anti-CD8a-PE (1:100, 53-6.7). For intracellular staining, cells were fixed; permeabilized with Fixation and Permeabilization Solution (BD Biosciences); washed three times; stained with anti-IFN-γ-Alexa Fluor 647 (1:100, XMG1.2), anti-Il-4-PE (1:100, 11B11), anti-Foxp3-PE (1:100, R16-715), anti-IL-10-PE (1:100, JES5-16E3), and anti-IL-12-FITC (1:100, C15.6) antibodies from BD Biosciences and anti-NOS2-APC (1:100, CXNFT) and anti-ARG1-Alexa Fluor 488 (1:100, A1exF5) antibodies from eBioscience for 0.5 h in the dark at 4 °C; and then subjected to flow cytometry analysis. Data were analysed with FlowJo software.

For TAM sorting, single cells prepared as described above were incubated with Fc-blocker and then stained with an APC-conjugated anti-F4/80 Ab (1:100, BD Biosciences, T45-2342) and a PE-Cy7-conjugated CD11b Ab (1:100, BD Biosciences, M1/70), followed by FACS to obtain TAMs.

### Arginase enzymatic activity

The enzymatic activity of arginase was determined using the Arginase Activity Assay Kit (MAK112, Sigma) according to the manufacturer’s protocol.

### ELISA and western blotting

For ELISA analysis, the indicated culture medium was collected, and the secreted levels of CCL22, IL-10, IL-6 and TNF-α were measured with ELISA kits according to the manufacturer’s instructions.

For western blot analysis, cells were harvested and lysed with RIPA buffer. The protein concentration was determined using a BCA kit. Samples were separated on 10% SDS-PAGE gels and blotted onto nitrocellulose membranes (Millipore). The following primary antibodies were incubated with the membranes at the indicated dilution: anti-ARG1 (1:1000, 93668, Cell Signaling Technology), anti-p-ERK1/2 (1:1000, 4370, Cell Signaling Technology), anti-ERK1/2 (1:1000, 4695, Cell Signaling Technology), anti-phosphorylated NF-κB p65 (S536) (1:1000, 3033, Cell Signaling Technology), anti-NF-κB p65 (1:1000, 8242, Cell Signaling Technology), anti-p-STAT3 (Y705) (1:1000, 9145, Cell Signaling Technology), anti-STAT3 (1:1000, 9139, Cell Signaling Technology), anti-METTL3 (1:1000, 195352, Abcam), anti-YTHDF1 (1:1000, 17479-1-AP, Proteintech), anti-SPRED2 (1:1000, S7320, Sigma) and anti-β-actin (1:5000, A5441, Sigma). The membranes were incubated with primary antibodies at 4 °C overnight, washed three times with TBST and then incubated with HRP-conjugated anti-mouse IgG (1:10,000, 7076, Cell Signaling Technology) or anti-rabbit IgG (1:10,000, 7074, Cell Signaling Technology) diluted in TBST containing 1% non-fat milk at room temperature for 1 h. After final washing with TBST, the membranes were developed by using ECL and visualized using a Tanon 5500.

### Immunohistochemistry

Mouse lungs and tumours were fixed in formalin, embedded, sectioned, and stained with HE or the indicated antibody as previously described^55^. The antibodies used were specific for CD3 (1:100, 5690, Abcam), CD4 (1:100, 183685, Abcam), CD8 (1:100, 98941, Cell Signaling Technology), FoxP3 (1:100, 12653, Cell Signaling Technology), F4/80 (1:100, 6640, Abcam), NOS2 (1:100, 15323, Abcam) and CD206 (1:100, 64693, Abcam). Sections were visualized, and images were acquired using a microscope (Olympus).

### Chromatin immunoprecipitation assay

Chromatin immunoprecipitation analysis of p-STAT3 was performed using the SimpleChIP Enzymatic Chromatin IP Kit (Cell Signaling Technology) according to the manufacturer’s instructions. The primer sequences used for ChIP analysis are listed in Supplementary Table 3.

### Quantitative real-time PCR

Total RNA was isolated from cells using TRIzol (Thermo Fisher Scientific). For qRT-PCR analyses of mRNAs, first-strand cDNA was synthesized using a PrimeScript qRT-PCR kit (Takara). The expression levels of target genes were examined with specific primers. The primers used are listed in Supplementary Table 3.

### Single-cell RNA seq (scRNA-seq) analysis

Tumours from WT or KO mice were digested and stained with an anti-CD45 antibody and 7-AAD. Live CD45+ cells were sorted by flow cytometry, washed, mixed with 10X Genomics Chromium single-cell RNA master mix and loaded onto a 10X Chromium chip to obtain single-cell cDNA following the manufacturer’s protocol. Libraries were subsequently prepared and sequenced on an Illumina NovaSeq 6000 instrument (Genergy Inc.).

Data were demultiplexed using CellRanger software based on 10X sample indexes, and paired-end FASTQ files were generated. Low-quality cells, doublets and potentially dead cells were removed. Cells with reads comprising less than or equal to 10% mitochondrial genes and more than or equal to 200 detected genes were collected for subsequent analyses. The reads were aligned to the mouse UCSC mm10 reference genome. Normalization, dimensionality reduction and clustering of single cells were also performed by Seurat. t-distributed stochastic neighbour embedding (t-SNE) was used for data visualization in two dimensions by using the top 30 significant principal components. Differentially expressed genes between each group of cells and the other groups of cells were identified by the Seurat-Bimod statistical test. The TopGO R package was used for Gene Ontology enrichment analysis for these significant differentially expressed genes. KEGG pathway enrichment analysis was performed using the hypergeometric test in R. Significantly enriched GO terms and KEGG pathways were also selected with the threshold *P*-value ≤ 0.05.

### m6A sequencing and quantification of mRNA methylation with m6A-IP and RT-qPCR (meRIP-qPCR)

Total RNA was isolated from cells using TRIzol (Thermo Fisher Scientific). Then, m6A sequencing was performed (CloudSeq Biotech, Shanghai, China). For meRIP-qPCR, poly (A) + mRNA was isolated using the Dynabeads mRNA Direct Purification Kit (61012, Thermo Fisher Scientific). We used 2 μg of purified RNA for m6A-containing mRNA enrichment using the Magna MeRIP m6A Kit (17-10499, Millipore), and the RNA was purified according to the manufacturer’s protocol. The resultant final product was used for qRT-PCR. The primers used for testing *Spred2* mRNA levels are listed in Supplementary Table 3.

### RNA methylation quantification

mRNA was isolated using the Dynabeads mRNA Direct Purification Kit (61012, Thermo Fisher Scientific). The methylation of the purified mRNA was quantified using the EpiQuik m6A RNA Methylation Quantification Kit (P-9005, EpiGentek) according to the manufacturer’s protocol.

### RNA immunoprecipitation-qPCR (RIP-qPCR)

This procedure was performed according to a protocol in a previously published report^26^. BMDMs were washed twice with PBS and lysed in lysis buffer (150 mM KCl, 10 mM HEPES pH 7.6, 2 mM EDTA, 0.5% NP-40, 0.5 mM dithiothreitol (DTT), 1:100 protease inhibitor cocktail and 400 U/ml RNase inhibitor). The cell lysates were centrifuged. A 50-μl aliquot of cell lysate was saved as the input, and the remaining sample was incubated with 20 µl of protein A beads previously bound to an IgG antibody or anti-YTHDF1 antibody (Proteintech) for 4 h at 4 °C. The beads were washed two times with wash buffer (50 mM Tris, 200 mM NaCl, 2 mM EDTA, 0.05% NP-40, 0.5 mM DTT, and an RNase inhibitor). RNA was eluted from the beads with 50 μl of RLT buffer and purified with Qiagen RNeasy columns. RNA was eluted in 100 µl of RNase-free water and reverse transcribed into cDNA using a PrimeScript qRT-PCR kit (Takara) according to the manufacturer’s instructions. The fold enrichment was detected by qRT-PCR. The primers used for testing *Spred2* mRNA levels are listed in Supplementary Table 3.

### Mouse tumour experiments

For tumour challenge experiments, 1 × 10^6^ LLC or B16 cells were injected subcutaneously into the back of age- and sex-matched mice. In the tumour cell and macrophage mixed culture model, BMDMs (2.5 × 10^5^) were mixed with B16 or LLC cells (1 × 10^6^) in 200 µL of PBS and co-injected subcutaneously into 6- to 8-week-old WT C57BL/6 mice. Tumour size was measured after injection. Tumour volume was calculated by using the following formula: *V* (mm^3^) = *a* × *b*^2^/2, where a and b indicate the long and short diameters, respectively. Tumours were then dissociated and subjected to IHC and FACS analyses. For survival studies, tumours were harvested when they reached 1.5 cm in any direction.

For the lung metastasis model, 2.5 × 10^5^ LLC cells or B16-luciferase cells in 200 µl of PBS were injected intravenously into mice. Mice bearing B16 tumours were monitored with an In Vivo Imaging System for fluorescence detection following substrate (D-luciferin) injection and anaesthetization with isoflurane. Data were analysed with LivingImage software.

### In vivo treatment

For Treg depletion, mice were injected via the tail vein with 250–500 µg of anti-CD25 (PC-61, BioXCell) monoclonal antibody following a protocol published previously^56^ with modifications. The anti-CD25 antibody was injected intravenously on days −2, 1, 4, 7 and 11. Harvested tumours were processed into single-cell suspensions with 1 mg/ml collagenase, 2.5 U/ml hyaluronidase and 0.1 mg/ml DNase.

For in vivo cancer therapy, B16 tumour-bearing mice were treated intravenously with an anti-PD-1 antibody (3 mg/kg, RMP1-14, BioXCell) on days 0, 3, 7, 11 and 15. Tumour volume and mouse survival were monitored following therapy.

### Macrophage depletion with clodronate liposomes

Macrophages were depleted using clodronate liposomes. In brief, macrophage depletion was performed by intravenous injection of 1 ml of liposomes per 100 g 48 h before the experiment. The mice were then injected with B16 cells (2.5 × 10^5^). Three injections of clodronate were given with a 3-day interval between each injection. Macrophage depletion was confirmed in pilot studies using flow cytometry.

### EdU injection

A stock solution of 10 mg/ml EdU (Sigma) was prepared in a normal saline solution (0.9%). For experiments, EdU (50 mg/kg) was injected intraperitoneally into mice, and tumour tissues were analysed after 2 days.

### mRNA stability analysis

To assess mRNA stability, BMDMs were treated with actinomycin D (Sigma) at a final concentration of 5 μg/mL for 0, 3 or 6 h. The cells were collected, and RNA samples were extracted for reverse transcription. mRNA transcript levels of interest were detected by qRT-PCR.

### Polysome profiling analysis

Polysome profiling analysis was performed as previously published^57^. BMDMs were treated with cycloheximide (0.1 mg/mL) for 3 min at room temperature to arrest and stabilize polysomes, washed with cold PBS, and then lysed in a cold room with 1 mL of polysome lysis buffer (0.3 M NaCl; 15 mM MgCl_2_.6H_2_O; 15 mM Tris-HCl, pH 7.4) supplemented with 10 µl of Triton X-100 (1% [v/v] final), 1 µl of 100 mg/ml cycloheximide (CHX) in DMSO (0.1 mg/ml final) and RNasin. The cell lysates were centrifuged at 17,000 × *g* for 15 min. The supernatants were fractionated with 10–50% sucrose gradients by centrifugation (200,000 × *g* for 1.5 h) in a Beckman ultracentrifuge. Each fraction of the density gradients was collected, and the absorbance was monitored at 260 nm. Ribosomal RNA content measured at 260 nm was plotted against the fraction number to obtain the polysome profile of each sample. RNA from each fraction was isolated and reverse transcribed. cDNA templates were amplified using real-time PCR analysis for polysome abundance on *Spred2* mRNA transcripts.

### Statistics and reproducibility

No statistical method was used to predetermine the sample size. Mice were assigned at random to treatment groups for all mouse studies and, where possible, mixed among cages. No mice were excluded from experiments. Blinded staining and blinded analysis were performed for IHC experiments. The data from independent experiments are shown as the mean ± SD. *P* values were calculated using two-sided *t*-tests by Microsoft excel 2016. For survival curves, the Gehan–Breslow–Wilcoxon test was used. Differences were considered statistically significant at *P ≤ 0.05, **P < 0.01 and ***P < 0.001. NS (non-significant) means *P* > 0.05.

### Reporting summary

Further information on research design is available in the Nature Research Reporting Summary linked to this article.

## Supplementary information

Supplementary Information

Reporting Summary

## Data Availability

The raw data from the MeRIP-Seq analysis of WT and KO BMDMs have been deposited in the Gene Expression Omnibus database under the accession code GEO: GSE146140. The scRNA-seq data analysed in this study are available under the accession identifier GSE159156. The remaining data are available within the Article, Supplementary Information or available from the authors upon request. Source data are provided with this paper.

## Source data

Source Data

## References

[1] Viola A, Sarukhan A, Bronte V, Molon B (2012). The pros and cons of chemokines in tumor immunology. Trends Immunol..

[2] Li J (2018). Tumor cell-intrinsic factors underlie heterogeneity of immune cell infiltration and response to immunotherapy. Immunity.

[3] Curiel TJ (2004). Specific recruitment of regulatory T cells in ovarian carcinoma fosters immune privilege and predicts reduced survival. Nat. Med..

[4] Facciabene A (2011). Tumour hypoxia promotes tolerance and angiogenesis via CCL28 and T(reg) cells. Nature.

[5] Amit I, Winter DR, Jung S (2016). The role of the local environment and epigenetics in shaping macrophage identity and their effect on tissue homeostasis. Nat. Immunol..

[6] Church SE, Galon J (2015). Tumor microenvironment and immunotherapy: the whole picture is better than a glimpse. Immunity.

[7] Colegio OR (2014). Functional polarization of tumour-associated macrophages by tumour-derived lactic acid. Nature.

[8] Noy R, Pollard JW (2014). Tumor-associated macrophages: from mechanisms to therapy. Immunity.

[9] Mantovani A, Marchesi F, Malesci A, Laghi L, Allavena P (2017). Tumour-associated macrophages as treatment targets in oncology. Nat. Rev. Clin. Oncol..

[10] Condeelis J, Pollard JW (2006). Macrophages: obligate partners for tumor cell migration, invasion, and metastasis. Cell.

[11] Wei W, Ji X, Guo X, Ji S (2017). Regulatory role of N(6)-methyladenosine (m(6)A) methylation in RNA processing and human diseases. J. Cell. Biochem..

[12] Zheng G (2013). ALKBH5 is a mammalian RNA demethylase that impacts RNA metabolism and mouse fertility. Mol. Cell.

[13] Yoon KJ (2017). Temporal control of mammalian cortical neurogenesis by m(6)A methylation. Cell.

[14] Haussmann IU (2016). m(6)A potentiates *Sxl* alternative pre-mRNA splicing for robust *Drosophila* sex determination. Nature.

[15] Lence T (2016). m(6)A modulates neuronal functions and sex determination in *Drosophila*. Nature.

[16] Batista PJ (2014). m(6)A RNA modification controls cell fate transition in mammalian embryonic stem cells. Cell Stem Cell.

[17] Wang Y (2014). N6-methyladenosine modification destabilizes developmental regulators in embryonic stem cells. Nat. Cell Biol..

[18] Lin S, Choe J, Du P, Triboulet R, Gregory RI (2016). The m(6)A methyltransferase METTL3 promotes translation in human cancer cells. Mol. Cell.

[19] Liu J, Eckert MA (2018). m(6)A mRNA methylation regulates AKT activity to promote the proliferation and tumorigenicity of endometrial cancer. Nat. Cell Biol..

[20] Zhang C (2016). Hypoxia induces the breast cancer stem cell phenotype by HIF-dependent and ALKBH5-mediated m(6)A-demethylation of NANOG mRNA. Proc. Natl Acad. Sci. USA.

[21] Visvanathan A (2018). Essential role of METTL3-mediated m(6)A modification in glioma stem-like cells maintenance and radioresistance. Oncogene.

[22] Zhang S (2017). m(6)A demethylase ALKBH5 maintains tumorigenicity of glioblastoma stem-like cells by sustaining FOXM1 expression and cell proliferation program. Cancer Cell.

[23] Li Z (2017). FTO plays an oncogenic role in acute myeloid leukemia as a N(6)-methyladenosine RNA demethylase. Cancer Cell.

[24] Vu LP (2017). The N(6)-methyladenosine (m(6)A)-forming enzyme METTL3 controls myeloid differentiation of normal hematopoietic and leukemia cells. Nat. Med..

[25] Ma JZ (2017). METTL14 suppresses the metastatic potential of hepatocellular carcinoma by modulating N(6) -methyladenosine-dependent primary MicroRNA processing. Hepatology.

[26] Han D (2019). Anti-tumour immunity controlled through mRNA m(6)A methylation and YTHDF1 in dendritic cells. Nature.

[27] Li HB (2017). m(6)A mRNA methylation controls T cell homeostasis by targeting the IL-7/STAT5/SOCS pathways. Nature.

[28] Pan W (2017). The DNA methylcytosine dioxygenase Tet2 sustains immunosuppressive function of tumor-infiltrating myeloid cells to promote melanoma progression. Immunity.

[29] Faget J (2011). Early detection of tumor cells by innate immune cells leads to T(reg) recruitment through CCL22 production by tumor cells. Cancer Res..

[30] Terzic J, Grivennikov S, Karin E, Karin M (2010). Inflammation and colon cancer. Gastroenterology.

[31] Kumar V (2016). CD45 phosphatase inhibits STAT3 transcription factor activity in myeloid cells and promotes tumor-associated macrophage differentiation. Immunity.

[32] Ullrich M (2018). OCD-like behavior is caused by dysfunction of thalamo-amygdala circuits and upregulated TrkB/ERK-MAPK signaling as a result of SPRED2 deficiency. Mol. Psychiatry.

[33] Zhao BS, Roundtree IA, He C (2017). Post-transcriptional gene regulation by mRNA modifications. Nat. Rev. Mol. Cell Biol..

[34] Barbieri I (2017). Promoter-bound METTL3 maintains myeloid leukaemia by m(6)A-dependent translation control. Nature.

[35] Buchmann K (2014). Evolution of innate immunity: clues from invertebrates via fish to mammals. Front. Immunol..

[36] Ginhoux F, Schultze JL, Murray PJ, Ochando J, Biswas SK (2016). New insights into the multidimensional concept of macrophage ontogeny, activation and function. Nat. Immunol..

[37] Martinez FO, Gordon S (2014). The M1 and M2 paradigm of macrophage activation: time for reassessment. F1000Prime Rep..

[38] Murray PJ (2014). Macrophage activation and polarization: nomenclature and experimental guidelines. Immunity.

[39] Wang J, Yan S, Lu H, Wang S, Xu D (2019). METTL3 attenuates LPS-induced inflammatory response in macrophages via NF-κB signaling pathway. Mediators Inflamm..

[40] Liu Y (2019). The N(6)-methyladenosine (m(6)A)-forming enzyme METTL3 facilitates M1 macrophage polarization through the methylation of STAT1 mRNA. Am. J. Physiol. Cell Physiol..

[41] Ohkura T (2019). Spred2 regulates high fat diet-induced adipose tissue inflammation, and metabolic abnormalities in mice. Front. Immunol..

[42] Itakura J (2017). Spred2-deficiecy protects mice from polymicrobial septic peritonitis by enhancing inflammation and bacterial clearance. Sci. Rep..

[43] Brenner D, Blaser H, Mak TW (2015). Regulation of tumour necrosis factor signalling: live or let die. Nat. Rev. Immunol..

[44] Hu X, Ivashkiv LB (2009). Cross-regulation of signaling pathways by interferon-gamma: implications for immune responses and autoimmune diseases. Immunity.

[45] Ouchi N, Parker JL, Lugus JJ, Walsh K (2011). Adipokines in inflammation and metabolic disease. Nat. Rev. Immunol..

[46] Mauer J (2014). Signaling by IL-6 promotes alternative activation of macrophages to limit endotoxemia and obesity-associated resistance to insulin. Nat. Immunol..

[47] Grivennikov SI, Greten FR, Karin M (2010). Immunity, inflammation, and cancer. Cell.

[48] Gabrilovich DI, Nagaraj S (2009). Myeloid-derived suppressor cells as regulators of the immune system. Nat. Rev. Immunol..

[49] Geiger R (2016). L-arginine modulates T cell metabolism and enhances survival and anti-tumor activity. Cell.

[50] Pesce JT (2009). Arginase-1-expressing macrophages suppress Th2 cytokine-driven inflammation and fibrosis. PLoS Pathog..

[51] El Kasmi KC (2008). Toll-like receptor-induced arginase 1 in macrophages thwarts effective immunity against intracellular pathogens. Nat. Immunol..

[52] Faust N, Varas F, Kelly LM, Heck S, Graf T (2000). Insertion of enhanced green fluorescent protein into the lysozyme gene creates mice with green fluorescent granulocytes and macrophages. Blood.

[53] Liu J (2020). N (6)-methyladenosine of chromosome-associated regulatory RNA regulates chromatin state and transcription. Science.

[54] Weischenfeldt, J. & Porse, B. Bone marrow-derived macrophages (BMM): isolation and applications. *CSH Protoc.* **2008**, 10.1101/pdb.prot5080 (2008). 10.1101/pdb.prot5080 21356739

[55] Wang L (2014). c-Myc-mediated epigenetic silencing of MicroRNA-101 contributes to dysregulation of multiple pathways in hepatocellular carcinoma. Hepatology.

[56] Wang X (2017). Cancer-FOXP3 directly activated CCL5 to recruit FOXP3(+)Treg cells in pancreatic ductal adenocarcinoma. Oncogene.

[57] Hubstenberger A (2017). P-body purification reveals the condensation of repressed mRNA Regulons. Mol. Cell.

