# Supplementary material for: RNA m6A methylation orchestrates cancer growth and metastasis via macrophage reprogramming
Source: Nat Commun. 2021 Mar 2;12:1394. doi: 10.1038/s41467-021-21514-8 (PMC7925544; doi:10.1038/s41467-021-21514-8)
Supplement: Supplementary file 1 — Supplementary Information [file 41467_2021_21514_MOESM1_ESM.pdf]

SUPPLEMENTARY INFORMATION

RNA m6A methylation orchestrates cancer growth and metastasis via macrophage reprogramming

Huilong Yin [1,2,3,4,9], Xiang Zhang [3,9], Pengyuan Yang [5], Xiaofang Zhang [3], Yingran Peng [3], Da Li [3], Yanping Yu [6], Ye Wu [3], Yidi Wang [7], Jinbao Zhang [3], Xiaochen Ding [8], Xiangpeng Wang [2,4], Angang Yang [1,2,4] ✉ and Rui Zhang [1,3] ✉

[1] The State Key Laboratory of Cancer Biology, Department of Immunology, Fourth Military Medical University, Xi'an, Shaanxi 710032, China

[2] Henan Key Laboratory of immunology and targeted therapy, School of Laboratory Medicine, Xinxiang Medical University, Xinxiang, Henan 453003, China

[3] The State Key Laboratory of Cancer Biology, Department of Biochemistry and Molecular Biology, Fourth Military Medical University, Xi'an, Shaanxi 710032, China

[4] Henan Collaborative Innovation Center of Molecular Diagnosis and Laboratory Medicine, School of Laboratory Medicine, Xinxiang Medical University, Xinxiang, Henan 453003, China

[5] Key Laboratory of Infection and Immunity of CAS, CAS Center for Excellence in Biomacromolecules, Institute of Biophysics, University of Chinese Academy of Sciences, Chinese Academy of Sciences, Beijing, 100101, China.

[6] The Second Ward of Gynecological Tumor, Shaanxi Provincial Tumor Hospital, Xi'an, Shaanxi, China

[7] Department of Thyroid, Breast and Vascular Surgery, Xijing Hospital, Fourth Military Medical University, Xi'an, China

[8] Department of Experimental Surgery, Xijing Hospital, Fourth Military Medical University, Xi'an, Shaanxi, China

[9] These authors contributed equally: Huilong Yin, Xiang Zhang

✉ Correspondence:

Prof. Angang Yang

The State Key Laboratory of Cancer Biology, Department of Immunology, Fourth Military Medical University,

No.169, Changle West Road Xi'an, Shaanxi, 710032, China.

Email: agyang@fmmu.edu.cn

Tel: +86-29-84712275

Prof. Rui Zhang

The State Key Laboratory of Cancer Biology, Department of Immunology, Fourth Military Medical University,

No.169, Changle West Road Xi'an, Shaanxi, 710032, China.

Email: ruizhang@fmmu.edu.cn

Tel: +86-29-84712272

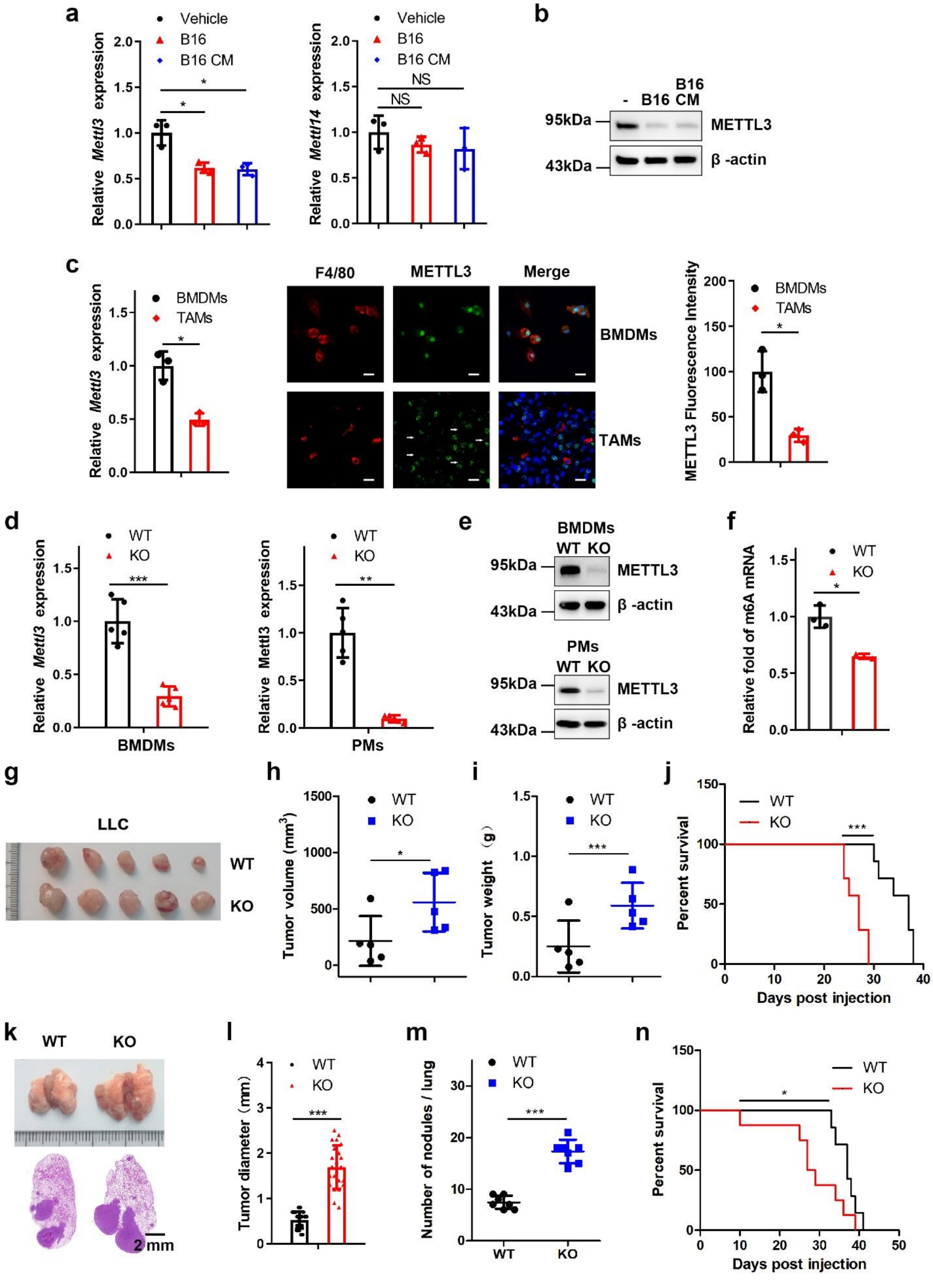
a
Vehicle
B16
B16 CM
Relative Mettl3 expression
Relative Mettl14 expression
NS
b
-
B16
B16 CM
95kDa
43kDa
METTL3
β -actin
c
BMDMs
TAMs
F4/80
METTL3
Merge
METTL3 Fluorescence Intensity
d
WT
KO
BMDMs
PMs
e
f
Relative fold of m6A mRNA
g
LLC
h
Tumor volume (mm3)
i
Tumor weight (g)
j
Percent survival
Days post injection
k
2 mm
l
Tumor diameter (mm)
m
Number of nodules / lung
n

Supplementary Figure 1 Myeloid-specific deletion of *Mettl3* promotes tumour growth and metastasis.

a,**b**, Expression of *Mettl3* and *Mettl14* was measured by qRT-PCR in WT BMDMs incubated with B16 cells or B16 cells culture medium (**a**), and western blotting was used to evaluate METTL3 expression (**b**). n=3 independent experiments (qRT-PCR). The blots are representative of n=3 independent experiments. **c**, Expression of *Mettl3* was determined in BMDMs and TAMs (two weeks after tumour cell injection) from B16 tumour-bearing WT mice by qRT-PCR (left panel) and immunofluorescence analysis (right panel). The white arrows highlight F4/80$^+$ cells. (n=3 mice per group). Scale bars, 20 μm. **d**,**e**, qRT-PCR (**d**) and western blotting (**e**) analysis of METTL3 in BMDMs and peritoneal macrophages (PMs) from WT and KO mice. n=5 mice per group for qRT-PCR. The blots are representative of n=3 independent experiments. **f**, The levels of m6A mRNA were analysis in WT and KO BMDMs. n=3 independent experiments. **g-j**, LLC cells were subcutaneously injected into WT and KO mice. Tumours were dissected and photographed (**g**). Tumour volume (**h**) (n=5 mice per group), tumour weights (**i**) (n=5 mice per group) and mouse survival (**j**) were recorded (n=7 mice per group). **k-n**, Representative images of tumours in mice injected with LLC cells through the tail vein. Representative HE staining pictures of lung sections were shown (**k**). Quantification of lung tumour diameter (**l**) (n=4 mice per group) and metastatic nodules (**m**) (n=7 mice per group). The survival of mice injected with LLC tumour cells through the tail vein was recorded (**n**) (n=7 mice for WT group and n=8 mice for KO group). Data are means ± SD. P values were determined by two-tailed t-test (**a, c, d, f, h, i, l** and **m**) and Gehan-Breslow-Wilcoxon Test (**j**, **n**). P ≤ 0.05 (*), P < 0.01 (**), and P < 0.001 (***). NS (non-significant) means P >0.05. The source data are provided as a Source Data file.

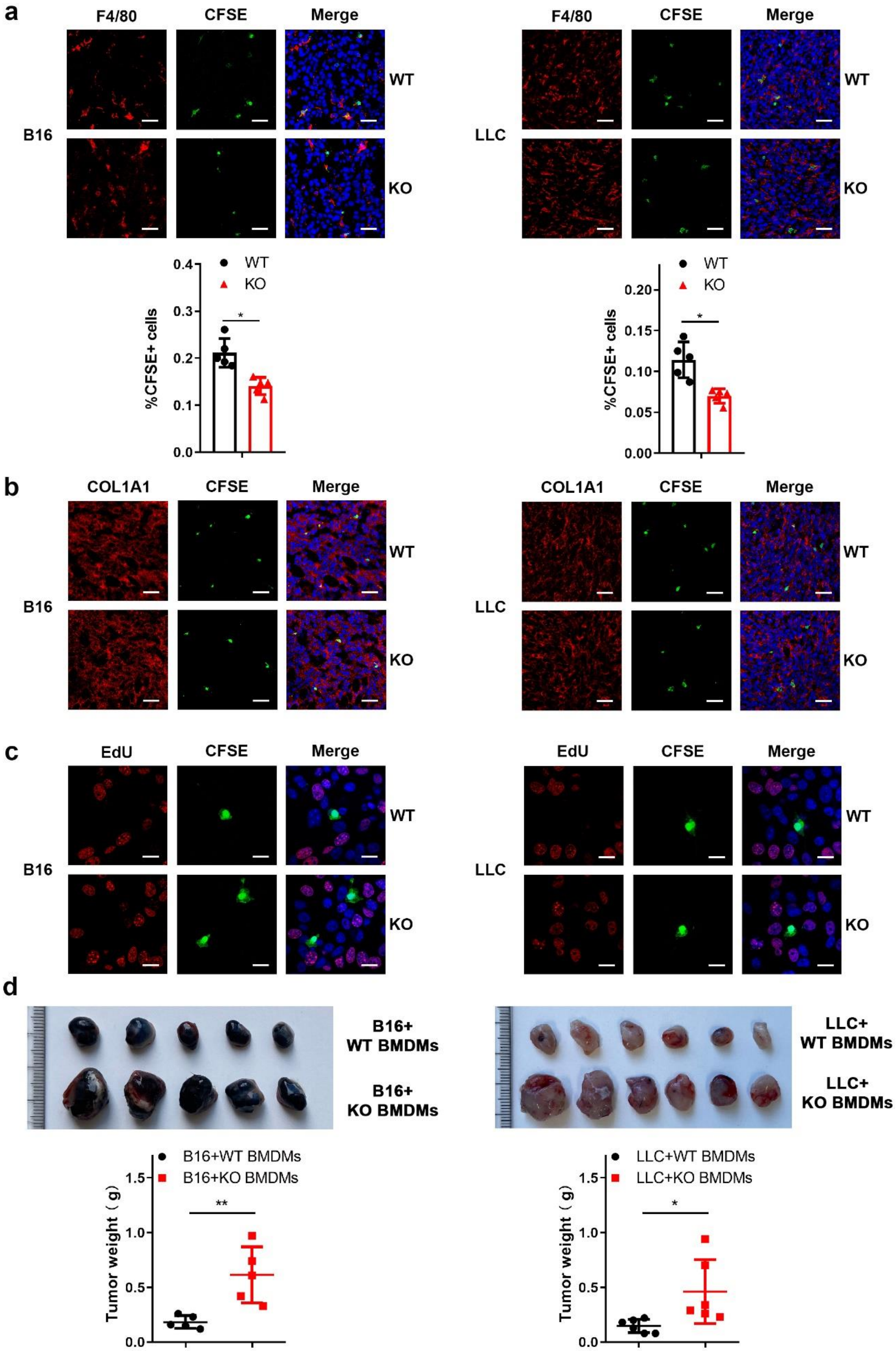
a
F4/80
CFSE
Merge
WT
KO
B16
LLC
%CFSE+ cells
WT
KO
b
COL1A1
CFSE
Merge
B16
LLC
WT
KO
c
EdU
CFSE
Merge
B16
LLC
WT
KO
d
B16+ WT BMDMs
B16+ KO BMDMs
LLC+ WT BMDMs
LLC+ KO BMDMs
Tumor weight（g）
B16+WT BMDMs
B16+KO BMDMs
LLC+WT BMDMs
LLC+KO BMDMs

Supplementary Figure 2 The KO BMDMs enhance tumour growth.

a-d, WT mice were subcutaneously inoculated with B16 or LLC cells mixed with CFSE-labeled WT or KO macrophages. Tumours were dissected 14 days after inoculation and co-stained with F4/80 (**a**) or COL1A1 (**b**) (n=5 mice per group). Scale bars, 40 μm. CFSE-positive macrophage proliferation was detected in B16 or LLC tumours using EdU proliferation markers (**c**). The bar graphs are representative of n=3 independent experiments. Scale bars, 10 μm. The tumours dissected were photographed and tumour weight were recorded **(d**) (n=5 mice for B16 and 6 mice for LLC per group). Data are means ± SD. P values were determined by two-tailed t-test (**a, d**). $P \leq 0.05$ (*) and $P < 0.01$ (**). The source data are provided as a Source Data file.

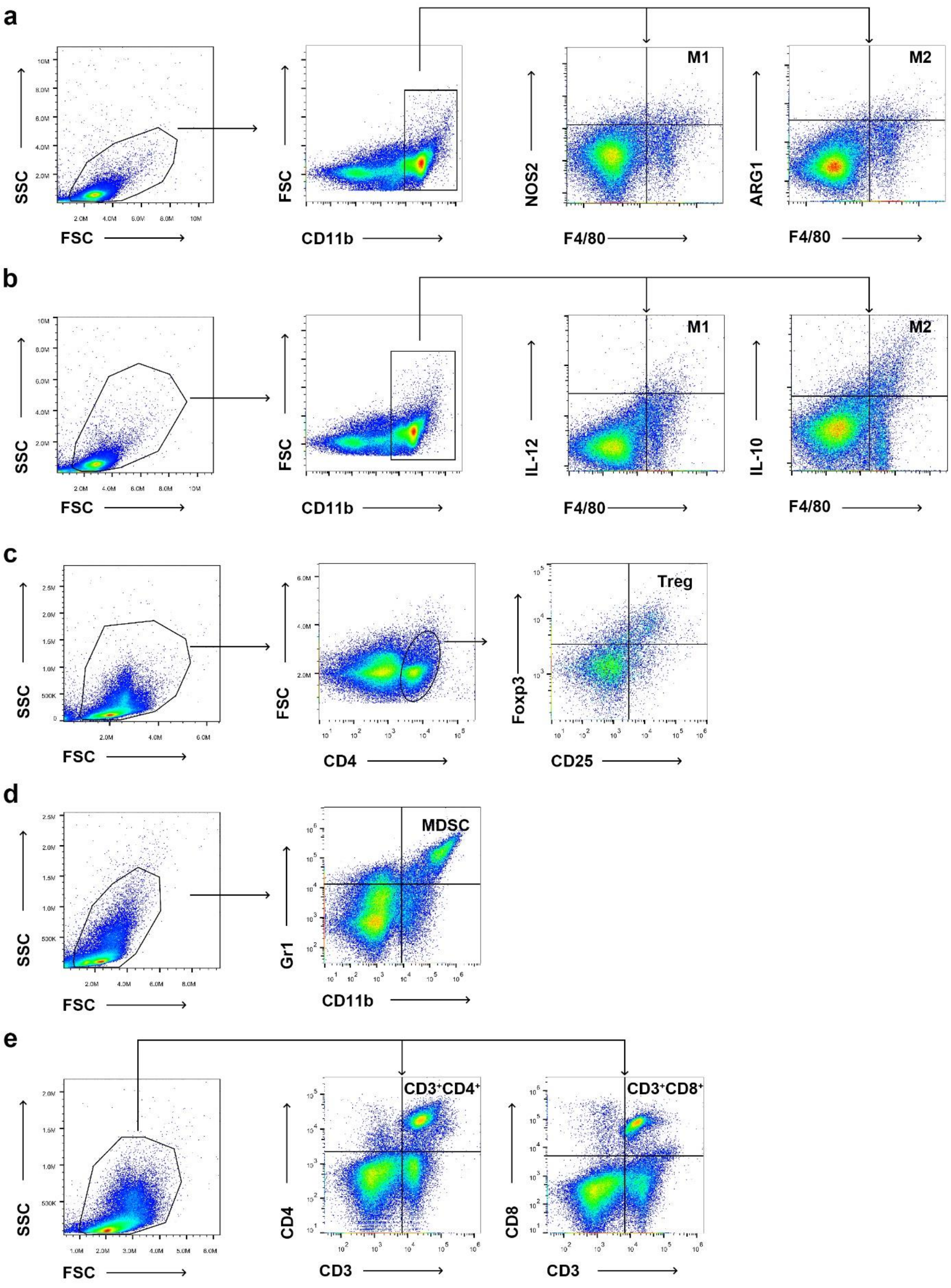
a
b
c
d
e
M1
M2
Treg
MDSC
CD3+CD4+
CD3+CD8+
SSC
FSC
CD11b
NOS2
ARG1
F4/80
IL-12
IL-10
CD4
Foxp3
CD25
Gr1
CD3
CD8

Supplementary Figure 3 Gating strategies used for cell sorting. a, Gating strategy to sort M1 macrophages ($F4/80^{+}NOS2^{high}$) and M2 macrophages ($F4/80^{+}ARG1^{high}$) cells from mice on Fig. S4a, S6a. **b**, Gating strategy to sort M1 macrophages ($F4/80^{+}IL\text{-}12^{high}$) and M2 macrophages ($F4/80^{+}IL\text{-}10^{high}$) cells from mice on Fig. S4b, S6a. **c**, Gating strategy to sort Treg ($CD4^{+}CD25^{+}Foxp3^{+}$) cells from mice on Fig. 3d, S4c, S6b. **d**, Gating strategy to sort MDSC ($CD11b^{+}Gr1^{+}$) cells from mice on Fig. S4d, S6b. **e**, Gating strategy to sort $CD3^{+}CD4^{+}$ T cells and $CD3^{+}CD8^{+}$ T cells from mice on Fig. S4e, S6c, S6d.

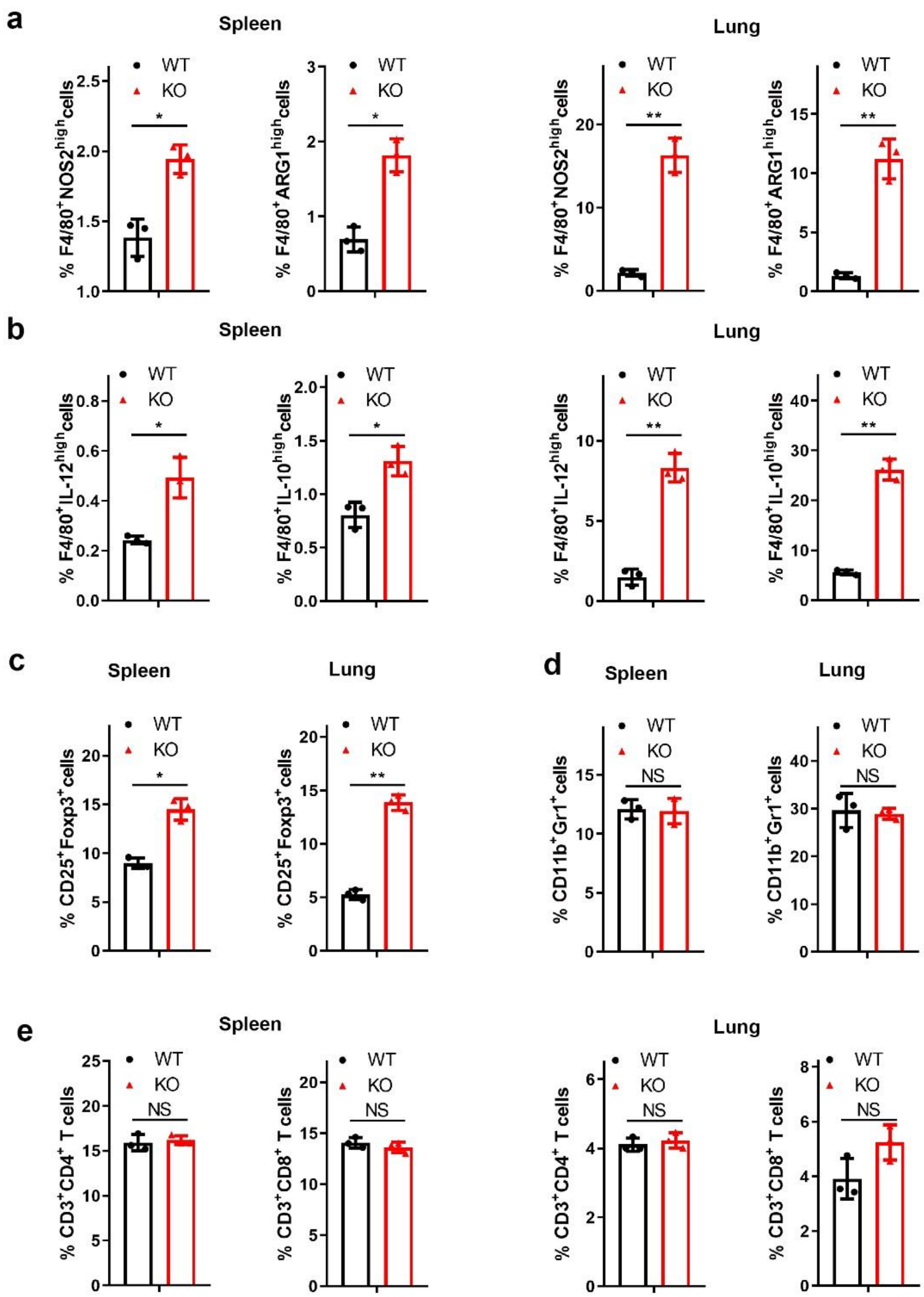

Supplementary Figure 4 *Mettl3* depletion in macrophages changes the immune microenvironment in spleen and lung.

a, M1 macrophages (F4/80^{+}NOS2^{high}) and M2 macrophages (F4/80^{+}ARG1^{high}) were determined by flow cytometry in spleen and lung, with CD11b^{+} cells gated. Percentages were shown as

numbers in quadrants and histogram. (n=3 mice per group). **b**, M1 macrophages (F4/80^{+}IL-12^{high}) and M2 macrophages (F4/80^{+}IL-10^{high}) were determined by flow cytometry in spleen and lung, with CD11b^{+} cells gated. Percentages were shown as numbers in quadrants and histogram. (n=3 mice per group). **c**, The percentage of Treg (CD25^{+}Foxp3^{+}) cells was determined by flow cytometry in spleen and lung, with CD4^{+} cells gated. Percentages were shown as numbers in quadrants and histogram. (n=3 mice per group). **d**, The percentage of CD11b^{+}Gr1^{+} MDSC cells was determined by flow cytometry in spleen and lung of WT and KO mice. (n=3 mice per group). **e**, The percentages of CD3^{+}CD4^{+} T cells and CD3^{+}CD8^{+} T cells were determined by flow cytometry in spleen and lung of WT and KO mice. (n=3 mice per group). Data are shown as the mean ± SD. P values were determined by two-tailed t-test (**a-e**). $P \leq 0.05$ (*) and $P < 0.01$ (**). NS (non-significant) means $P > 0.05$. The source data are provided as a Source Data file.

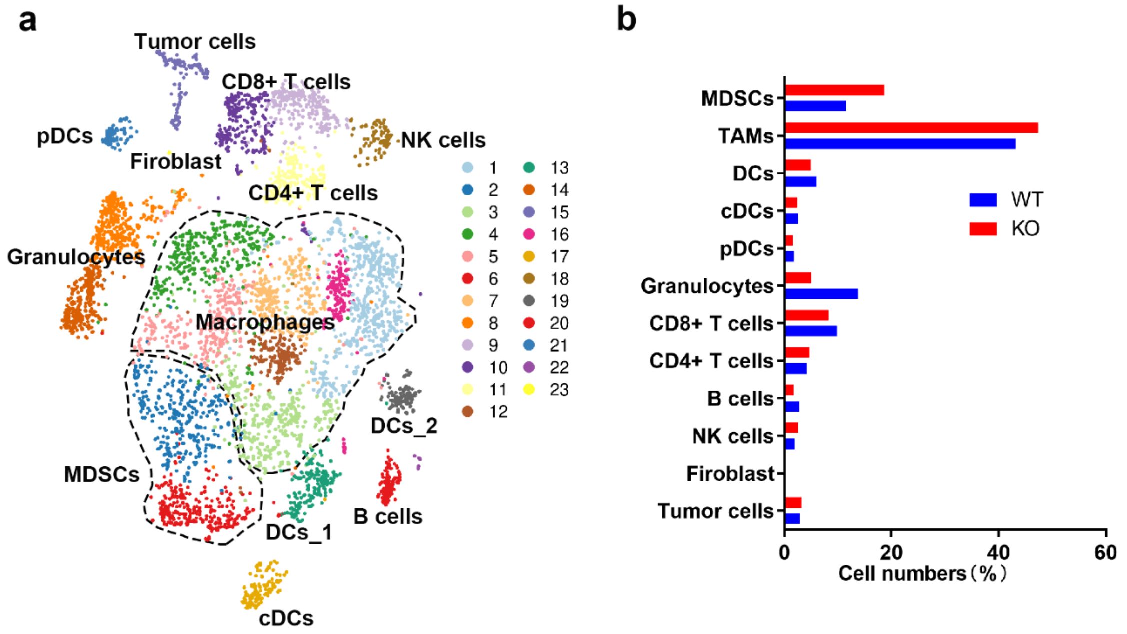

Supplementary Figure 5 Immune infiltration profiling in B16 tumours with scRNA-Seq.

a, The CD45^{+} immune-infiltrated cells in B16 tumours were enriched. Then, pooled samples of five mice per group were analysed by scRNA-seq. Distinct clusters of immune cells were identified according to expression signatures. The colors denote the distinct clusters of cells. **b**, The frequency of cells in each cluster was shown.

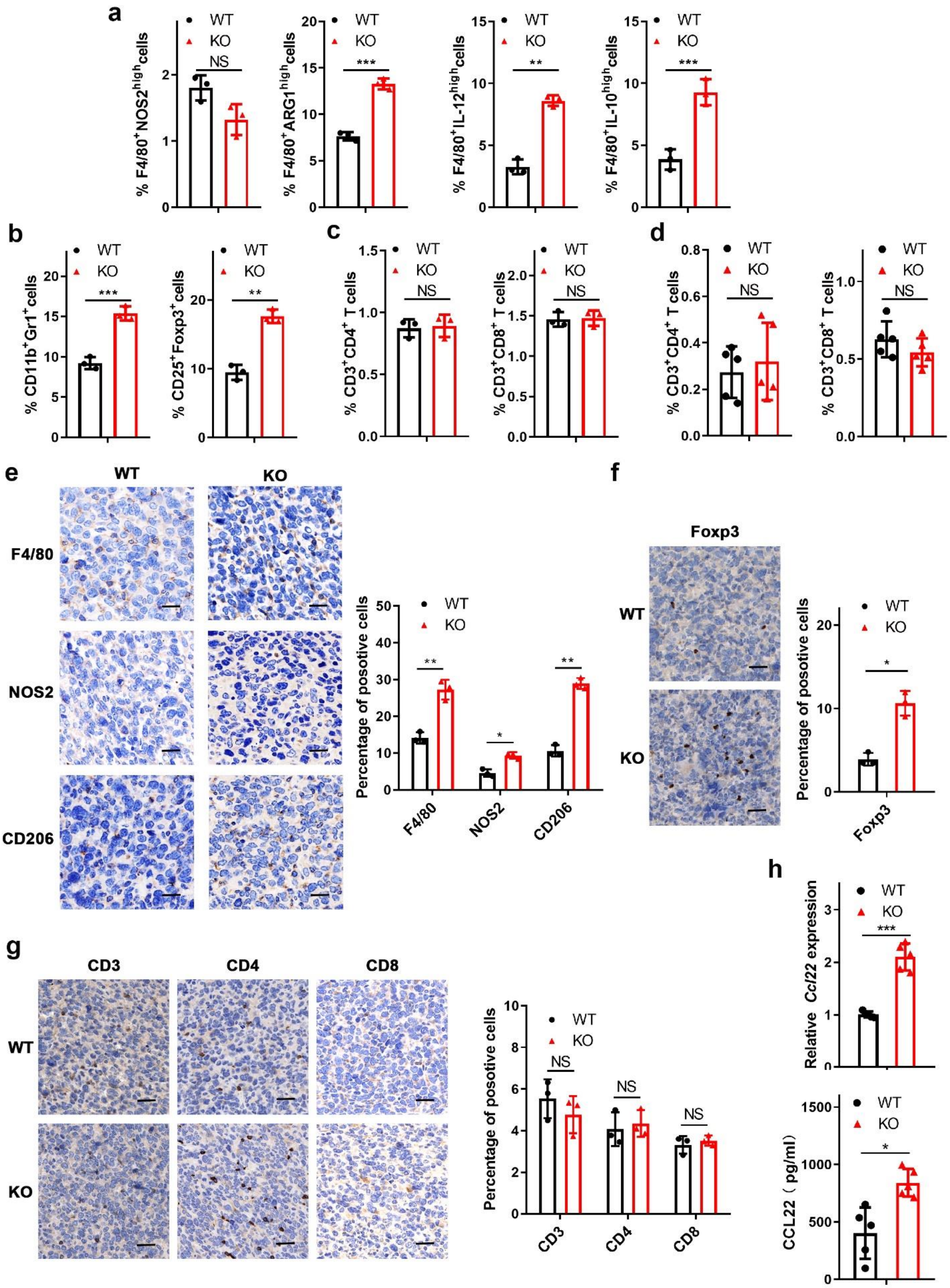
a
b
c
d
e
f
g
h
WT
KO
NS
***
**
*
% F4/80+NOS2high cells
% F4/80+ARG1high cells
% F4/80+IL-12high cells
% F4/80+IL-10high cells
% CD11b+Gr1+ cells
% CD25+Foxp3+ cells
% CD3+CD4+ T cells
% CD3+CD8+ T cells
F4/80
NOS2
CD206
Foxp3
CD3
CD4
CD8
Percentage of posotive cells
Relative Ccl22 expression
CCL22（pg/ml)

Supplementary Figure 6 *Mettl3* depletion in macrophages reshapes the tumour microenvironment by enhancing M1 and M2-like TAMs and Treg infiltration

a, Flow cytometry analysis of macrophage subpopulations of the LLC tumours from tumour-bearing mice. The percentages of ($F4/80^{+}NOS2^{high}$) M1-like, ($F4/80^{+}ARG1^{high}$) M2-like, ($F4/80^{+}IL\text{-}12^{high}$) M1-like and ($F4/80^{+}IL\text{-}10^{high}$) M2-like TAMs were calculated, with $CD11b^{+}$ cells gated. (n=3 mice per group). **b**, LLC tumours were dissected and digested to obtain single-cell suspensions. Infiltrating Treg ($CD25^{+}Foxp3^{+}$) and MDSC ($CD11b^{+}Gr1^{+}$) cells were fluorescent stained and analysed by flow cytometry, with $CD4^{+}$ cells gated. (n=3 mice per group). **c**, The percentages of $CD3^{+}CD4^{+}$ T cells and $CD3^{+}CD8^{+}$ T cells in LLC tumours were calculated. (n=3 mice per group). **d**, The percentages of $CD3^{+}CD4^{+}$ T cells and $CD3^{+}CD8^{+}$ T cells were determined by flow cytometry in B16 tumours of WT and KO mice. (n=5 mice per group). **e**, **f**, Immunohistochemical staining of macrophage markers (**e**) and Treg marker (**f**) in tumours of mice. (n=3 mice per group). Scale bars: 30μm.**g**, Immunohistochemical staining of immune infiltrate T cells markers in LLC tumours of mice. (n=3 mice per group). Scale bars: 30μm. **h**, qRT-PCR (left panel) and ELISA (right panel) analysis of CCL22 in WT and KO BMDMs. (n=5 mice per group). Data are shown as the mean ± SD. P values were determined by two-tailed t-test (**a-h**). $P \leq 0.05$ (*), $P < 0.01$ (**), and $P < 0.001$ (***). NS (non-significant) means $P > 0.05$. The source data are provided as a Source Data file.

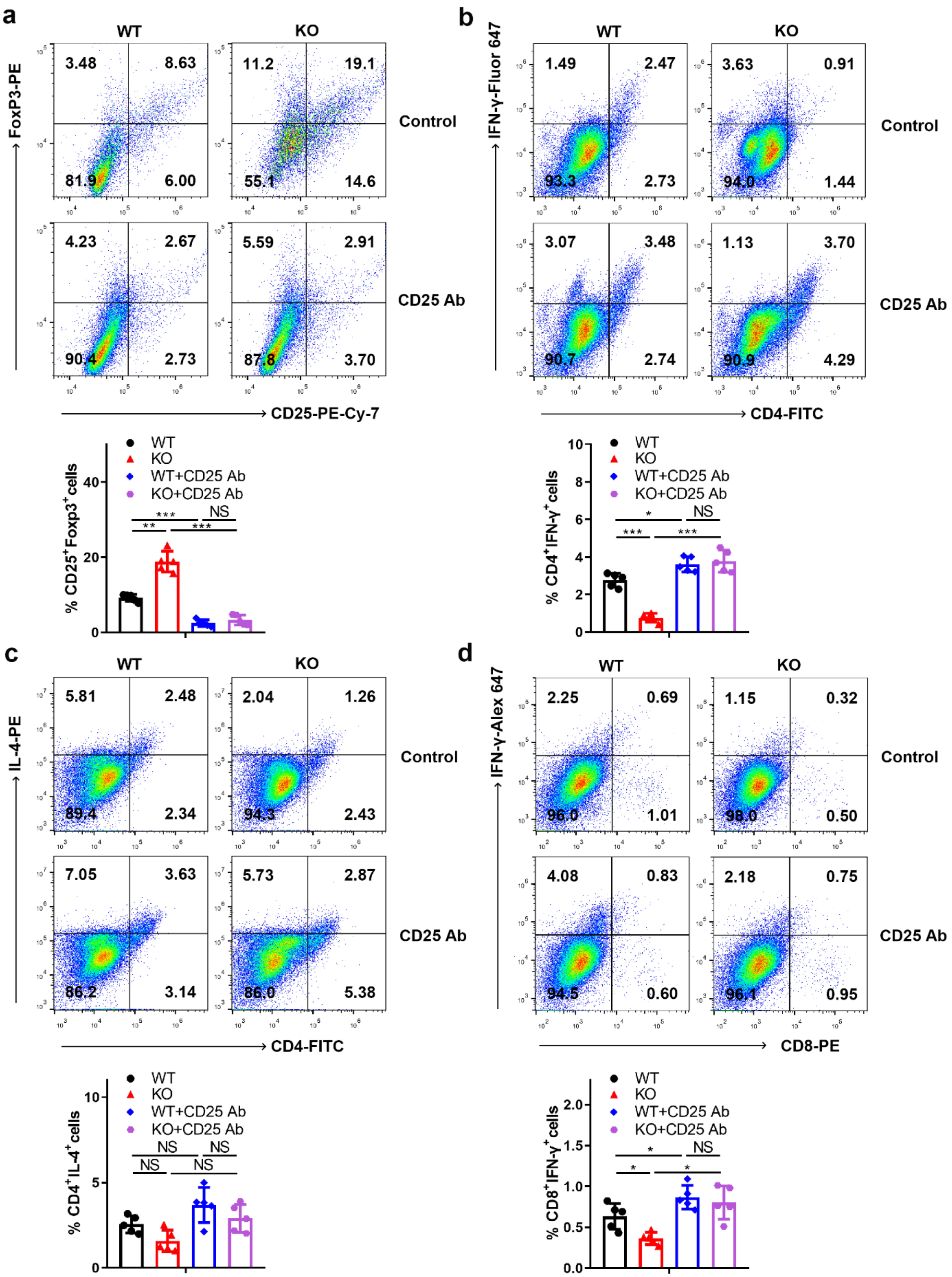
a
WT
KO
FoxP3-PE
3.48
8.63
81.9
6.00
11.2
19.1
55.1
14.6
Control
4.23
2.67
90.4
2.73
5.59
2.91
87.8
3.70
CD25 Ab
CD25-PE-Cy-7
% CD25+Foxp3+cells
WT
KO
WT+CD25 Ab
KO+CD25 Ab
***
**
NS
***
b
IFN-γ-Fluor 647
1.49
2.47
93.3
2.73
3.63
0.91
94.0
1.44
3.07
3.48
90.7
2.74
1.13
3.70
90.9
4.29
CD4-FITC
% CD4+IFN-γ+cells
*
***
NS
c
IL-4-PE
5.81
2.48
89.4
2.34
2.04
1.26
94.3
2.43
7.05
3.63
86.2
3.14
5.73
2.87
86.0
5.38
% CD4+IL-4+cells
d
IFN-γ-Alex 647
2.25
0.69
96.0
1.01
1.15
0.32
98.0
0.50
4.08
0.83
94.5
0.60
2.18
0.75
96.1
0.95
CD8-PE
% CD8+IFN-γ+cells

Supplementary Figure 7 The impact of anti-CD25 antibody on T cells activation in B16 tumours.

The efficacy of the anti-CD25 antibody to deplete Treg was tested in B16 tumour bearing mice. And the frequencies of Treg (**a**), Th1 (**b**), Th2 (**c**) and IFN-γ-secreting $CD8^+$ T (**d**) cells were determined by flow cytometry (n=5 mice per group). Data are means ± SD. P values were determined by two-tailed t-test (**a-d**). $P \leq 0.05$ (*), $P < 0.01$ (**), and $P < 0.001$ (***). NS (non-significant) means $P > 0.05$. The source data are provided as a Source Data file.

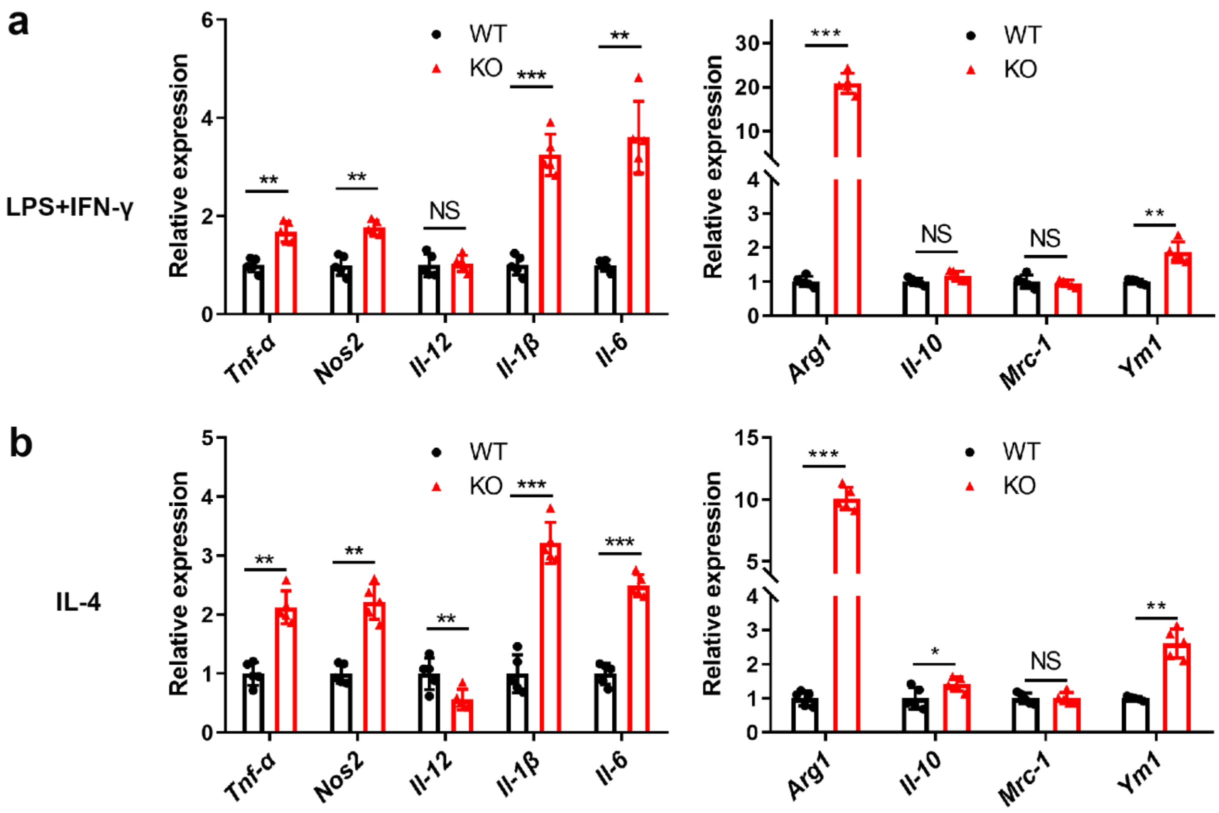

Supplementary Figure 8 ***Mettl3*** **depletion promotes M1 and M2 polarization of BMDMs.**

a,**b**, qRT-PCR analysis of M1 and M2 macrophages-associated gene expression in WT and KO BMDMs treated with mouse recombinant LPS (50 ng/mL) + IFN-γ (40 ng/mL) (**a**) or IL-4 (40 ng/mL) (**b**) for 24h. (n=5 mice per group). Data are means ± SD. P values were determined by two-tailed t-test (**a**,**b**). $P \leq 0.05$ (*), $P < 0.01$ (**), and $P < 0.001$ (***). NS (non-significant) means $P > 0.05$. The source data are provided as a Source Data file.

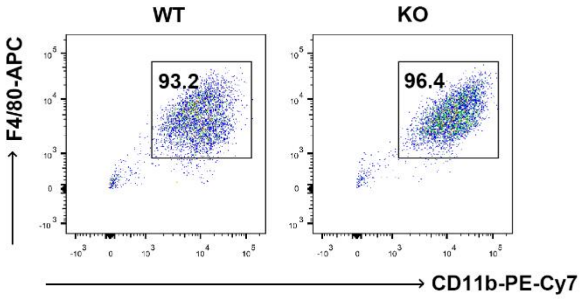

Supplementary Figure 9 the purity of TAMs tested by flow cytometry was shown.

The TAMs in B16 tumours from WT or KO mice were sorted and the purity of TAMs was tested by flow cytometry.

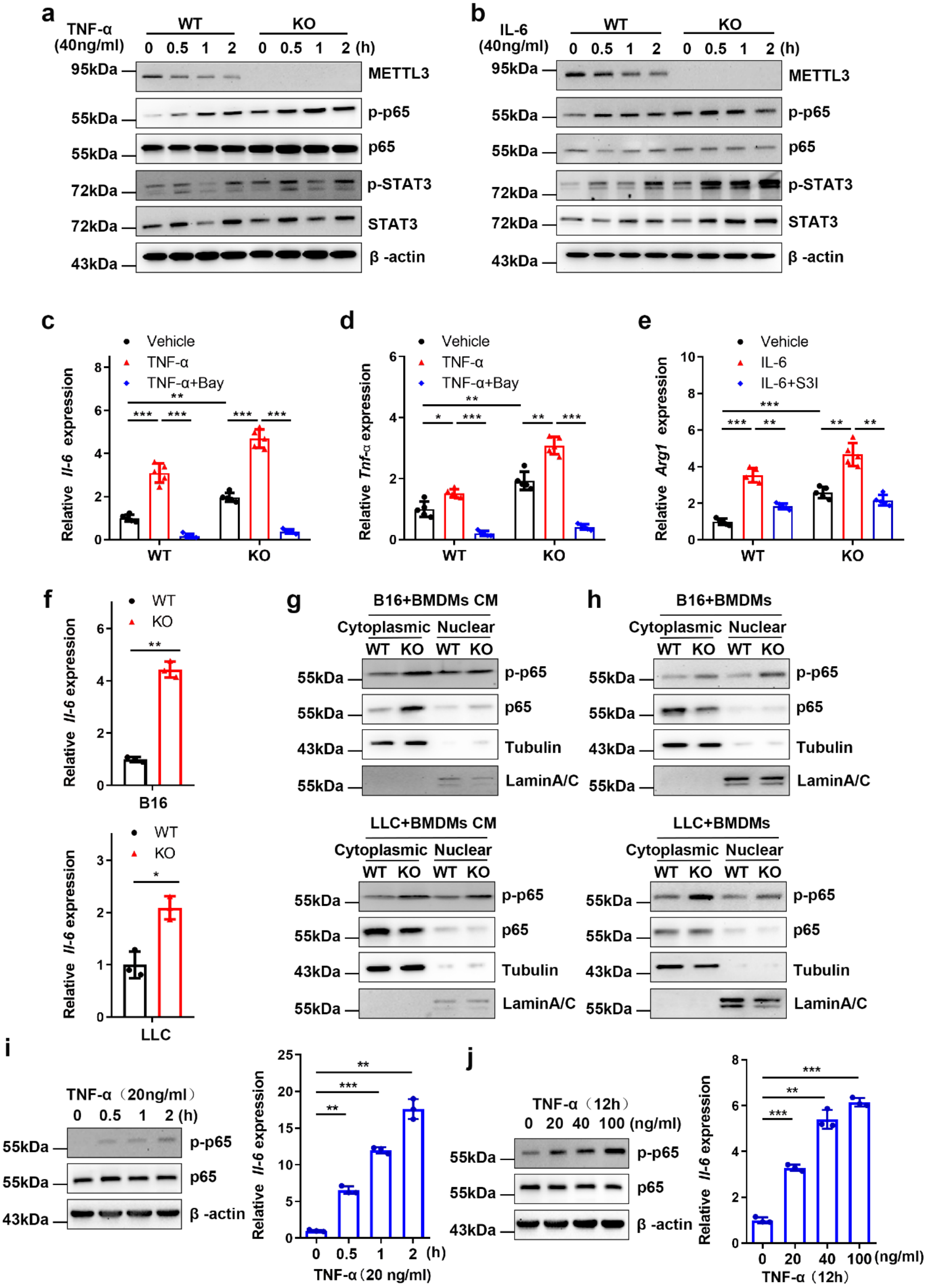

a
TNF-α (40ng/ml)
WT
KO
0 0.5 1 2 0 0.5 1 2 (h)
95kDa
METTL3
55kDa
p-p65
55kDa
p65
72kDa
p-STAT3
72kDa
STAT3
43kDa
β -actin
b
IL-6 (40ng/ml)
WT
KO
0 0.5 1 2 0 0.5 1 2 (h)
95kDa
METTL3
55kDa
p-p65
55kDa
p65
72kDa
p-STAT3
72kDa
STAT3
43kDa
β -actin
c
Vehicle
TNF-α
TNF-α+Bay
Relative Il-6 expression
WT
KO
d
Vehicle
TNF-α
TNF-α+Bay
Relative Tnf-α expression
WT
KO
e
Vehicle
IL-6
IL-6+S3I
Relative Arg1 expression
WT
KO
f
WT
KO
Relative Il-6 expression
B16
LLC
g
B16+BMDMs CM
LLC+BMDMs CM
Cytoplasmic
Nuclear
WT KO WT KO
p-p65
p65
Tubulin
LaminA/C
h
B16+BMDMs
LLC+BMDMs
Cytoplasmic
Nuclear
WT KO WT KO
p-p65
p65
Tubulin
LaminA/C
i
TNF-α（20ng/ml）
0 0.5 1 2 (h)
p-p65
p65
β -actin
Relative Il-6 expression
TNF-α（20 ng/ml)
j
TNF-α（12h）
0 20 40 100 (ng/ml)
p-p65
p65
β -actin
Relative Il-6 expression
TNF-α（12h）

Supplementary Figure 10 Macrophages with *Mettl3* depletion regulates cytokine responses by communicating with tumour cells.

a,**b**, Stimulation with cytokines TNF-α (**a**) and IL-6 (**b**) induced p-p65 and p-STAT3 expression in a time-dependent manner in WT and KO BMDMs. The blots are representative of n=3 independent experiments. **c**,**d**, WT and KO BMDMs were treated with TNF-α or TNF-α+BAY-11-7082, followed by qRT-PCR analysis of *Il-6* (**c**) and *Tnf-α* (**d**) expression (n=5 mice per group). **e**, WT and KO BMDMs were treated with IL-6 or IL-6+S3I-201, followed by qRT-PCR analysis of *Arg1* expression (n=5 mice per group). **f**, qRT-PCR analysis of *Il-6* expression levels in B16 (upper panel) and LLC cells (lower panel) incubated with WT or KO BMDMs culture medium (50% of the final culture medium) for 24 hours. n=3 independent experiments. **g**, B16 (upper panel) and LLC (lower panel) cells were treated with WT or KO BMDMs culture medium for 24h. Cytoplasmic and nuclear proteins were separated. Western blotting was used to evaluate p-p65 expression in the cytoplasm and nucleus. The blots are representative of n=3 independent experiments. **h**, B16 (upper panel) and LLC (lower panel) cells were cocultured with WT or KO BMDMs. Western blotting was used to test p-p65 expression in the cytoplasm and nucleus. The blots are representative of n=3 independent experiments. **i**, B16 cells were treated with TNF-α (20 ng/mL) at the indicated time points. Western blotting was used to analyse p-p65 expression (left panel). The blots are representative of n=3 independent experiments. Relative expression of *Il-6* was determined by qRT-PCR (right panel). n=3 independent experiments. **j**, B16 cells were treated with different concentrations of TNF-α for 12h. p-p65 expression was analysed by western blotting (left panel). The blots are representative of n=3 independent experiments. The relative expression of *Il-6* was determined by qRT-PCR (right panel). n=3 independent experiments. Data are means ± SD. P values were determined by two-tailed t-test (**c-f, i** and **j**). $P \leq 0.05$ (*), $P < 0.01$ (**), and

$P < 0.001$ (***). NS (non-significant) means $P > 0.05$. The source data are provided as a Source Data file.

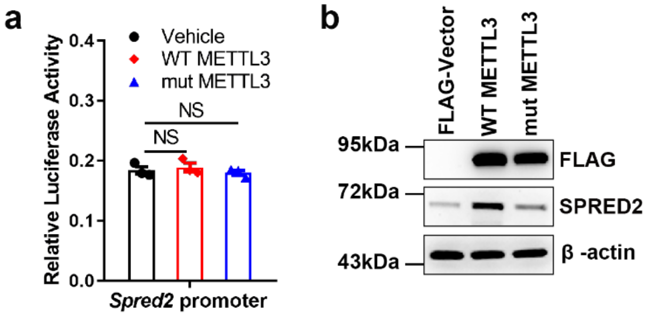

Supplementary Figure 11 METTL3 promotes SPRED2 translation independent of promoter binding.

a, Dual luciferase report assays showed that neither METTL3 wild-type (WT METTL3) nor the catalytically dead mutant of METTL3 (mut METTL3) bound to the Spred2 promoter. **b**, Western blot analysis of the indicated proteins in BMDMs overexpressing WT METTL3 and mut METTL3. The blots are representative of n=3 independent experiments. All quantitative data are means ± SD from three independent experiments. P values were determined by a two-tailed t-test (**a**). NS (non-significant) means P >0.05. The source data are provided as a Source Data file.

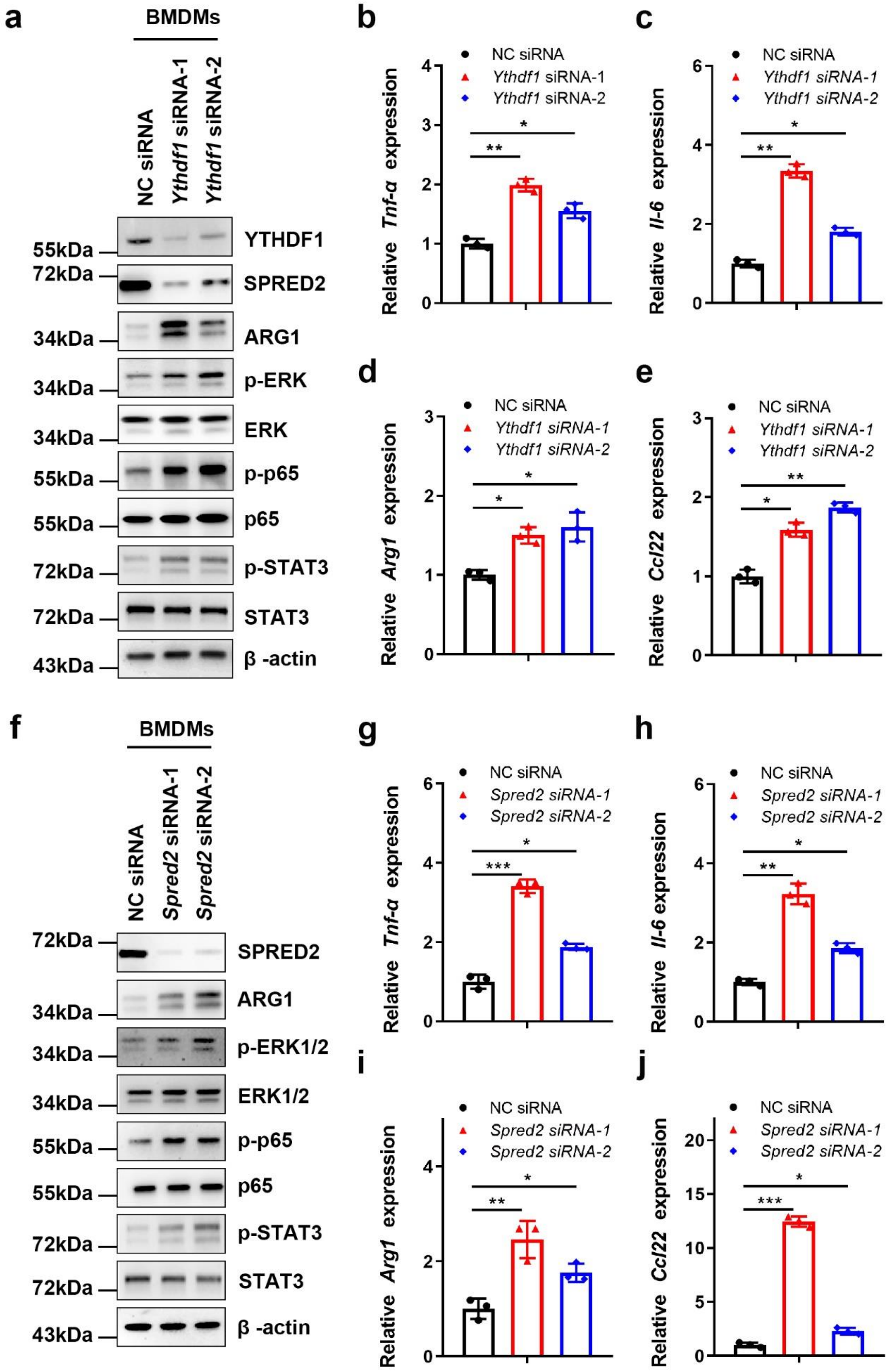
a
BMDMs
NC siRNA
Ythdf1 siRNA-1
Ythdf1 siRNA-2
YTHDF1
SPRED2
ARG1
p-ERK
ERK
p-p65
p65
p-STAT3
STAT3
β -actin
55kDa
72kDa
34kDa
43kDa
b
Relative Tnf-α expression
c
Relative Il-6 expression
d
Relative Arg1 expression
e
Relative Ccl22 expression
f
BMDMs
NC siRNA
Spred2 siRNA-1
Spred2 siRNA-2
SPRED2
ARG1
p-ERK1/2
ERK1/2
p-p65
p65
p-STAT3
STAT3
β -actin
g
h
i
j

Supplementary Figure 12 Knockdown of YTHDF1 or SPRED2 increases the expression of *Tnf-α*, *Il-6*, *Ccl22* and *Arg1* through NF-kB/STAT3.

a, Western blotting analysis of indicated proteins in BMDMs transfected with NC siRNA or *Ythdf1* siRNA. The blots are representative of n=3 independent experiments. **b-e**, qRT-PCR analysis of *Tnf-α* (**b**), *Il-6* (**c**), *Ccl22* (**d**) and *Arg1* (**e**) mRNA expression in BMDMs transfected with NC siRNA or *Ythdf1* siRNA. **f**, Western blotting analysis of protein expression in BMDMs transfected with NC siRNA or *Spred2* siRNA. The blots are representative of n=3 independent experiments. **g-j**, qRT-PCR analysis of *Tnf-α* (**g**), *Il-6* (**h**), *Ccl22* (**i**) and *Arg1* (**j**) mRNA expression in BMDMs transfected with NC siRNA or *Ythdf1* siRNA. All quantitative data are means ± SD from three independent experiments. P values were determined by two-tailed t-test (**b-e** and **g-j**). $P \leq 0.05$ (*), $P < 0.01$ (**), and $P < 0.001$ (***). The source data are provided as a Source Data file.

Supplementary Table 1. MAPK pathway related genes (294)

	Genes
**MAPK pathway related genes**	*Akt1, Akt2, Akt3, Angpt1, Angpt2, Angpt4, Araf, Areg, Arrb1, Arrb2, Atf2, Atf4, Bdnf, Braf, Cacna1a, Cacna1b, Cacna1c, Cacna1d, Cacna1e, Cacna1f, Cacna1g, Cacna1h, Cacna1i, Cacna1s, Cacna2d1, Cacna2d2, Cacna2d3, Cacna2d4, Cacnb1, Cacnb2, Cacnb3, Cacnb4, Cacng1, Cacng2, Cacng3, Cacng4, Cacng5, Cacng6, Cacng7, Cacng8, Casp3, Cd14, Cdc25b, Cdc42, Chuk, Crk, Crkl, Csf1, Csf1r, Daxx, Ddit3, Dusp1, Dusp10, Dusp16, Dusp2, Dusp3, Dusp4, Dusp5, Dusp6, Dusp7, Dusp8, Dusp9, Ecsit, Efna1, Efna2, Efna3, Efna4, Efna5, Egf, Egfr, Elk1, Elk4, Epha2, Erbb2, Erbb3, Erbb4, Ereg, Fas, Fasl, Fgf1, Fgf10, Fgf15, Fgf16, Fgf17, Fgf18, Fgf2, Fgf20, Fgf21, Fgf22, Fgf23, Fgf3, Fgf4, Fgf5, Fgf6, Fgf7, Fgf8, Fgf9, Fgfr1, Fgfr2, Fgfr3, Fgfr4, Flna, Flnb, Flnc, Flt1, Flt3, Flt3l, Flt4, Fos, Gadd45a, Gadd45b, Gadd45g, Gm5741, Gna12, Gng12, Grb2, Hgf, Hras, Hspa1a, Hspa1b, Hspa1l, Hspa2, Hspa8, Hspb1, Igf1, Igf1r, Igf2, Ikbkb, Ikbkg, Il1a, Il1b, Il1r1, Il1rap, Ins1, Ins2, Insr, Irak1, Irak4, Jun, Jund, Kdr, Kit, Kitl, Klk1b4, Kras, Lamtor3, Map2k1, Map2k2, Map2k3, Map2k4, Map2k5, Map2k6, Map2k7, Map3k1, Map3k11, Map3k12, Map3k13, Map3k14, Map3k2, Map3k3, Map3k4, Map3k5, Map3k6, Map3k7, Map3k8, Map4k1, Map4k2, Map4k3, Map4k4, Mapk1, Mapk10, Mapk11, Mapk12, Mapk13, Mapk14, Mapk3, Mapk7, Mapk8, Mapk8ip1, Mapk8ip2, Mapk8ip3, Mapk9, Mapkapk2, Mapkapk3, Mapkapk5, Mapt, Max, Mecom, Mef2c, Met, Mknk1, Mknk2, Mras, Myc, Myd88, Nf1, Nfatc1, Nfatc3, Nfkb1, Nfkb2, Ngf, Ngfr, Nlk, Nr4a1, Nras, Ntf3, Ntf5, Ntrk1, Ntrk2, Pak1, Pak2, Pdgfa, Pdgfb, Pdgfc, Pdgfd, Pdgfra, Pdgfrb, Pgf, Pla2g4a, Pla2g4b, Pla2g4c, Pla2g4d, Pla2g4e, Pla2g4f, Ppm1a, Ppm1b, Ppp3ca, Ppp3cb, Ppp3cc, Ppp3r1, Ppp3r2, Ppp5c, Prkaca, Prkacb, Prkca, Prkcb, Prkcg, Ptpn5, Ptpn7, Ptprr, Rac1, Rac2, Rac3, Raf1, Rap1a, Rap1b, Rapgef2, Rasa1, Rasa2, Rasgrf1, Rasgrf2, Rasgrp1, Rasgrp2, Rasgrp3, Rasgrp4, Rela, Relb, Rps6ka1, Rps6ka2, Rps6ka3, Rps6ka4, Rps6ka5, Rps6ka6, Rras, Rras2, Sos1, Sos2, Spred2, Srf, Stk3, Stk4, Stmn1, Tab1, Tab2, Taok1, Taok2, Taok3, Tek, Tgfa, Tgf1, Tgfb2, Tgfb3, Tgfbr1, Tgfbr2, Tnf, Tnfrsf1a, Tradd, Traf2, Traf6, Trp53, Vegfa, Vegfb, Vegfc, Vegfd.*

Supplementary Table 2. siRNA sequences used in experiments

	sense（5'-3'）	antisense（5'-3'）
Spred2-siRNA-1	GCGAGCGACAGAAAGACAATT	UUGUCUUUCUGUCGCUCGCTT
Spred2-siRNA-2	GCUACGGACAGUUCUUCUATT	UAGAAGAACUGUCCGUAGCTT
Ythdf1-siRNA-1	CCCGUAUCUCACUACCUAUTT	AUAGGUAGUGAGAUACGGGTT
Ythdf1-siRNA-2	GGACAUUGGUACUUGGGAUTT	AUCCCAAGUACCAAUGUCCTT

Supplementary Table 3. qRT-PCR primers

Mettl3-F	CTGGGCACTTGGATTTAAGGAA
Mettl3-R	TGAGAGGTGGTGTAGCAACTT
Mettl14-F	CTGAGAGTGCGGATAGCATTG
Mettl14-R	GAGCAGATGTATCATAGGAAGCC
Ccl22-F	AGGTCCCTATGGTGCCAATGT
Ccl22-R	CGGCAGGATTTTGAGGTCCA
Ythdf1-F	ACAGTTACCCCTCGATGAGTG
Ythdf1-R	GGTAGTGAGATACGGGATGGGA
Il-12-F	GGAAGCACGGCAGCAGAATA
Il-12-R	AACTTGAGGGAGAAGTAGGAATGG
Nos2-F	GCAGAGATTGGAGGCCTTGTG
Nos2-R	GGGTTGTTGCTGAACTTCCAGTC
Tnf-α-F	CAGGAGGGAGAACAGAAACTCCA
Tnf-α-R	CCTGGTTGGCTGCTTGCTT
Il-6-F	CCACTTCACAAGTCGGAGGCTTA
Il-6-R	GCAAGTGCATCATCGTTGTTCATAC
Il-1β-F	TCCAGGATGAGGACATGAGCAC
Il-1β-R	GAACGTCACACACCAGCAGGTTA
Arg1-F	AGACAGCAGAGGAGGTGAAGAG
Arg1-R	CGAAGCAAGCCAAGGTTAAAGC
Il-10-F	CCCTTTGCTATGGTGTCCTT
Il-10-R	TGGTTTCTCTTCCCAAGACC
Ym1-F	CATTCAGTCAGTTATCAGATTCC
Ym1-R	AGTGAGTAGCAGCCTTGG
Mrc-F	AAACACAGACTGACCCTTCCC
Mrc-R	GTTAGTGTACCGCACCCTCC
actin-F	GAGACCTTCAACACCCCAGC
actin-R	ATGTCACGCACGATTTCCC
ChIP-*Arg1*-F	CATGATTCCAAAAATGAGATTTTCC
ChIP-*Arg1*-R	GTTGTTATTATTATTATTATTTACAGGAAGGC
m6A-IP-*Spred2*-F	GAAGAAACACACCCGGACGAT
m6A-IP-*Spred2*-R	CTCCTTCCTGTGGGAACCAT
